# Role of MCP-1 and IL-8 in viral anterior uveitis, and contractility and fibrogenic activity of trabecular meshwork cells

**DOI:** 10.1038/s41598-021-94391-2

**Published:** 2021-07-22

**Authors:** Jiyoung Lee, Jin A. Choi, Hyun-hee Ju, Ju-Eun Kim, Soon-Young Paik, Ponugoti Vasantha Rao

**Affiliations:** 1 Department of Ophthalmology and Visual Science, College of Medicine, St. Vincent’s Hospital, The Catholic University of Korea, Banpo-daero 222, Seocho-gu, Seoul, 137-701 Republic of Korea; 2 Department of Ophthalmology, Duke University School of Medicine, Durham, NC USA; 3 Department of Microbiology, College of Medicine, The Catholic University of Korea, Seoul, Republic of Korea; 4 Department of Pharmacology and Cancer Biology, Duke University School of Medicine, Durham, NC USA

**Keywords:** Cytoskeleton, Mechanisms of disease, Inflammation, Glaucoma

## Abstract

The inflammatory chemokines, monocyte chemoattractant protein (MCP)-1 and IL-8, are produced by normal trabecular meshwork cells (TM) and elevated in the aqueous humor of primary open angle glaucoma (POAG) and hypertensive anterior uveitis associated with viral infection. However, their role in TM cells and aqueous humor outflow remains unclear. Here, we explored the possible involvement of MCP-1 and IL-8 in the physiology of TM cells in the context of aqueous outflow, and the viral anterior uveitis. We found that the stimulation of human TM cells with MCP-1 and IL-8 induced significant increase in the formation of actin stress fibers and focal adhesions, myosin light chain phosphorylation, and the contraction of TM cells. MCP-1 and IL-8 also demonstrated elevation of extracellular matrix proteins, and the migration of TM cells. When TM cells were infected with HSV-1 and CMV virus, there was a significant increase in cytoskeletal contraction and Rho-GTPase activation. Viral infection of TM cells revealed significantly increased expression of MCP-1 and IL-8. Taken together, these results indicate that MCP-1 and IL-8 induce TM cell contractibility, fibrogenic activity, and plasticity, which are presumed to increase resistance to aqueous outflow in viral anterior uveitis and POAG.

## Introduction

Glaucoma is the second most common cause of irreversible blindness, resulting from the acquired loss of the retinal ganglion cells. While elevated intraocular pressure (IOP) is a major risk factor for glaucoma, the precise mechanisms causing retinal ganglion cells death are not fully understood. During the past decade, a growing body of literature indicates that inflammatory component plays a role in the pathogenesis of glaucoma^1,2^. Involvement of T-cell mediated cytotoxicity, autoantibodies, the complement cascade, and innate immunity with inflammasome activation have been documented in the neurodegeneration in glaucoma^1^. Especially, pro-inflammatory molecules such as tumor necrosis factor alpha (TNF-α), interleukins (IL), chemokines, C-reactive protein (CRP), and serum amyloid A have been shown to be up-regulated in glaucoma^2^.

Ocular inflammation leads to increased outflow resistance in various ways. First, acute inflammation may result in the intertrabecular spaces to be obstructed by inflammatory material^3^, and resultant excessive phagocytosis can cause cell migration away from the trabecular meshwork (TM), which eventually decrease the TM cell density^4^. With chronic inflammation, aqueous outflow could be obstructed from scarring of the juxtacanalicular tissue. The inflammatory damage to the TM cells cumulative and irreversible loss of juxtacanalicular cells could contribute to an increased risk of IOP elevation in uveitic eyes^5^. Recently, research attention has been directed toward the inflammatory cytokines / chemokines in the aqueous humor (AH) of the glaucomatous eyes^6–8^. Increased levels of TGF-β, IL-8 and monocyte chemoattractant protein (MCP)-1 have been found to be consistently elevated in the AH of patients with primary open angle glaucoma (POAG)^8–17^. In addition, the levels of MCP-1 and IL-8 reportedly have been shown to have a positive correlation with the levels of IOP^13^. These inflammatory cytokines may decrease cellularity of the TM cells by inducing cell migration away from the outflow tract or possibly exert a direct cytotoxic effect^4,18^. While TGF-β is well characterized as a cardinal cytokine in the molecular mechanisms regulating AH outflow^19^, relatively little is known about other inflammatory chemokines regarding their mechanism in the aqueous outflow pathway.

The role of inflammatory mediators is crucial in the pathogenesis of uveitic glaucoma, in which inflammation triggered by infection, injury or an autoimmune disease is a major contributor in the pathogenesis. Among the etiologies of inflammation, virus infection has been increasingly implicated as one of the important causes of hypertensive anterior uveitis, which includes Posner–Schlossman syndrome (PSS) and Fuchs uveitis syndrome (FUS)^20^. The most common viruses associated with hypertensive anterior uveitis include herpes simplex virus (HSV)-1, cytomegalovirus (CMV), and varicella zoster virus (VZV)^21^. Hypertensive anterior uveitis is characterized by suddenly elevated IOP with low grade inflammation, which usually subsides with the cessation of inflammation^20,21^. In the AH of patients with PSS and FUS, the inflammatory chemokines including MCP-1 and IL-8 were reportedly to be elevated than in controls^22–24^, displaying higher levels than in POAG cases^25^.

When a literature search was conducted from PubMed updated until June 2020 for studies involved measuring levels of MCP-1 and IL-8 in the AH of patients undergoing surgical procedure, numerous studies in the recent 10 years have demonstrated that levels of MCP-1 and IL-8 are elevated in the AH of patients with POAG and hypertensive anterior uveitis such as Posner-Schlossman syndrome and Fuchs’ heterochromic uveitis (Supplementary Table S1), in which HSV-1 and CMV infection is an important causative factor.

MCP-1 and IL-8 are both pro-inflammatory chemokines. MCP-1 is a member of the CC class of the chemokine (CC Chemokine Ligand 2), triggering chemotaxis and trans-endothelial migration of monocyte to inflammatory lesion, by interacting with the membrane CC chemokine receptor 2 (CCR2) in monocytes^26^. IL-8, also known as a neutrophil chemotactic factor, belongs to the CXC class, activating cell-surface receptors, CXCR1 and CXCR2^27^. It has been shown that MCP-1 and IL-8 could contribute not only to host defense but also to multiple inflammatory processes such as angiogenesis, cancer progression, and autoimmune diseases^26^. By binding the surface receptors of the target cells, chemokines exert their effect via activation of G-protein coupled receptors as well as the Rho family proteins to influence cell motility^26^. In support of the effects of MCP-1 and IL-8 on cytoskeletal contractile activity, Janjanam et al.^28^ reported that MCP-1 stimulates actin polymerization and cell migration in vascular smooth muscle cell. Lai et al.^29^ showed that IL-8 increases endothelial cell migration via phosphoinositide 3-Kinase-Rac1/RhoA Pathway. The signaling pathways of MCP-1 and IL-8 have been also recognized to be involved in epithelial-mesenchymal transition (EMT) of tumor cells^30^.

The human trabecular meshwork (TM), the key regulator of IOP, exhibits distinct patterns and behaviors, displaying typical characteristics of endothelium, fibroblast, smooth muscle, and macrophage^31^. Interestingly, normal TM cells constitutively secrete MCP-1 and IL-8^32^. In light of the current evidences of the elevation of these chemokines in glaucomatous eyes, these chemokines are presumed to play an important role in the homeostasis of AH outflow. However, we do not have sufficient knowledge regarding the role of MCP-1 and IL-8 in the TM cell physiology and AH outflow. To gain more insights not only into the MCP-1 and IL-8 signaling pathways in the TM cell physiology but also into their pathologic role in POAG and hypertensive anterior uveitis, we first explored the effects of MCP-1 and IL-8 on human TM cells in the context of AH outflow and IOP. Next, we investigated the expression of MCP-1 and IL-8 upon CMV or HSV-1 infection in human TM cells along with the expression of their receptors, CCR2 and CXCR1 in normal human TM cells.

## Results

### Contractile activity, actin cytoskeletal organization and adhesive characteristics in human TM cells induced by MCP-1 and IL-8

**Figure 1:**
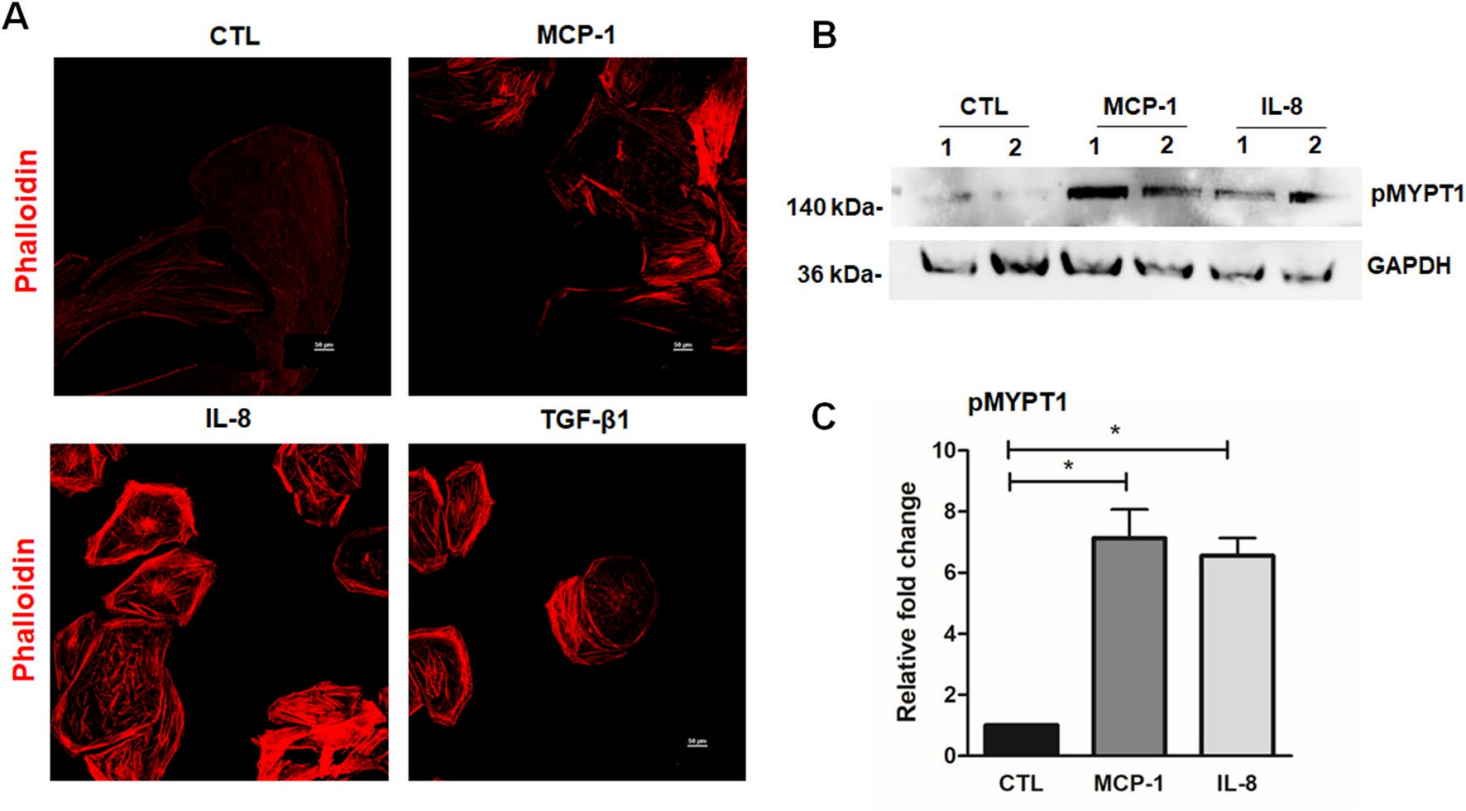
MCP-1 and IL-8 induces actin stress fibers and alters contractile properties of human TM cells. Primary cultured TM cells we generated were cultured on 2% gelatin-coated glass cover slips, serum-starved for 24 h and were treated with MCP-1 (100 ng/ml) or IL-8 (100 ng/ml) for 2 h or TGF-β 1 (10 ng/ml) for 24 h before staining for F-actin with TRITC-Phalloidin. Representative confocal images of stained cells show an increase in actin stress fibers (red) with MCP-1 and IL-8 treatment (**A**). MCP-1 and IL-8 treatment also revealed a significant increase in the levels of phosphorylated MYPT1 (**B**,**C**) relative to control (CTL) based on immunoblotting and densitometric analyses. Glyceraldehyde-3-phosphate dehydrogenase (GAPDH) was immunoblotted as a loading control for the cell lysates. The immunoblot data were normalized to GAPDH. Full-length blots/gels are presented in Supplementary Figure S1. *P < 0.05. N = 3, values are mean ± SEM and one-way ANOVA with Dunnett’s multiple comparison was done. *MCP-1* monocyte chemoattractant protein, *IL-8* interleukin-8, *MYPT1* myosin phosphatase targeting subunit 1, *GAPDH* glyceraldehyde 3-phosphate dehydrogenase.

Elevated level of MCP-1 and IL-8 in the AH of patients with POAG as well as those with hypertensive anterior uveitis, which is characterized by sudden abrupt elevation of IOP (Supplementary Table S1), led us to test a possible role for MCP-1 and IL-8 in TM cell actin cytoskeletal organization and focal adhesions formation. To address this aspect, we initially examined MCP-1 and IL-8 mediated changes in actin stress fibers and MLC (myosin light chain) phosphorylation, which is a key regulator of myosin II-driven contractile activity in human TM cells. Serum-starved TM cells treated with MCP-1 (100 ng/ml) or IL-8 (100 ng/ml) for 2 h showed a robust and significant increase in phosphorylation of myosin phosphatase target subunit 1 (pMYPT1), an essential regulator of cellular contractility and actomysin interaction^33^, in immunoblotting and densitometric analysis (Fig. 1B,C; uncropped images are shown in supplementary Fig S1), with associated increase in actin stress fibers, being comparable to TM cells treated with TGF-β1 (Fig. 1A).

**Figure 2:**
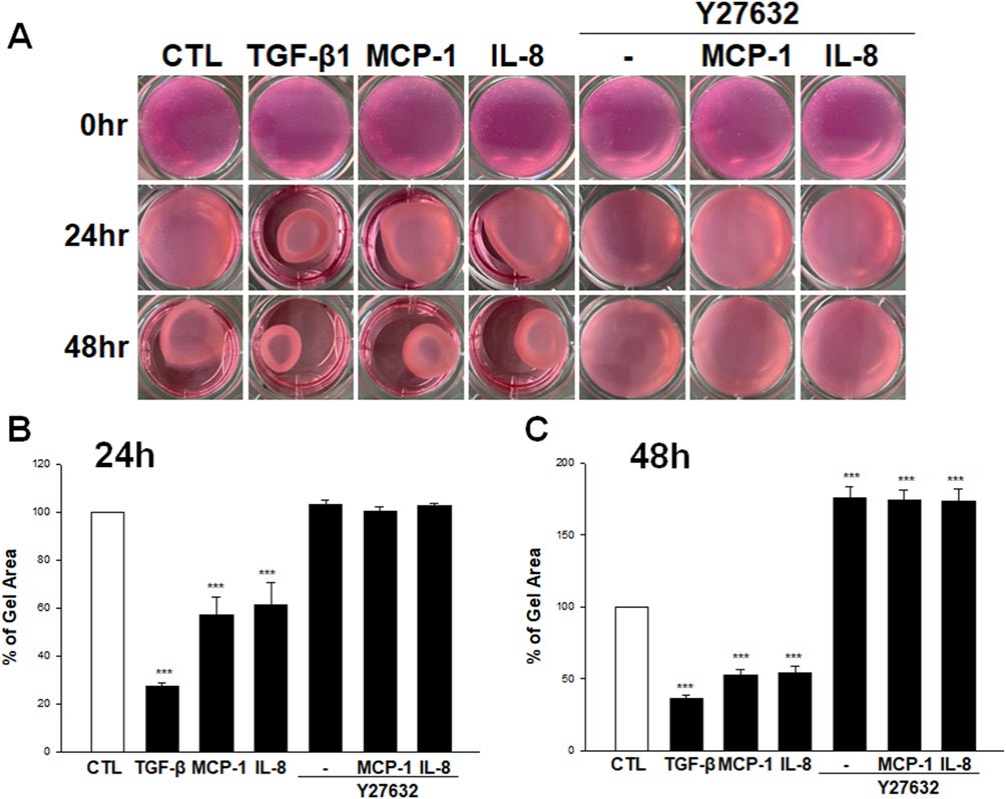
The effect of MCP-1 and IL-8 on human TM cell contraction using collagen gel contraction assay. After serum-starvation for 24 h, primary TM cells obtained from ScienCell Research Labs were embedded in collagen gels and treated with MCP-1 (100 ng/ml), IL-8 (100 ng/ml) or TGF-β1 (15 ng/ml) alone or in the presence of Rho-kinase inhibitor-Y27632 (10 µM for 30 min pretreatment) or with inhibitors alone for 24 h and 48 h. (**A**) At 24 h, collagen gels were significantly contracted by MCP-1, IL-8, and TGF-β1 treatment (*P* < 0.001) compared with the control group. However, pre-treatment with Y-27632 (10 µM) prevented the contraction by MCP-1 and IL-8. (**B**) After treatment with MCP-1, IL-8 or TGF-β1 for 48 h, MCP-1, IL-8, and TGFβ1 induced a further increase in the contraction of gels compared with the control group (*P* < 0.001, all), while the pre-treatment with Y-27632 significantly decreased contraction compared with the control group (*P* < 0.001) (**C**). The results of three independent experiments are expressed as the mean ± SEM, and one-way ANOVA with Dunnett’s multiple comparison was done. Culture wells were photographed (bottom) and area of gel matrices were measured using Image J software. ***P < 0.001.

**Figure 3:**
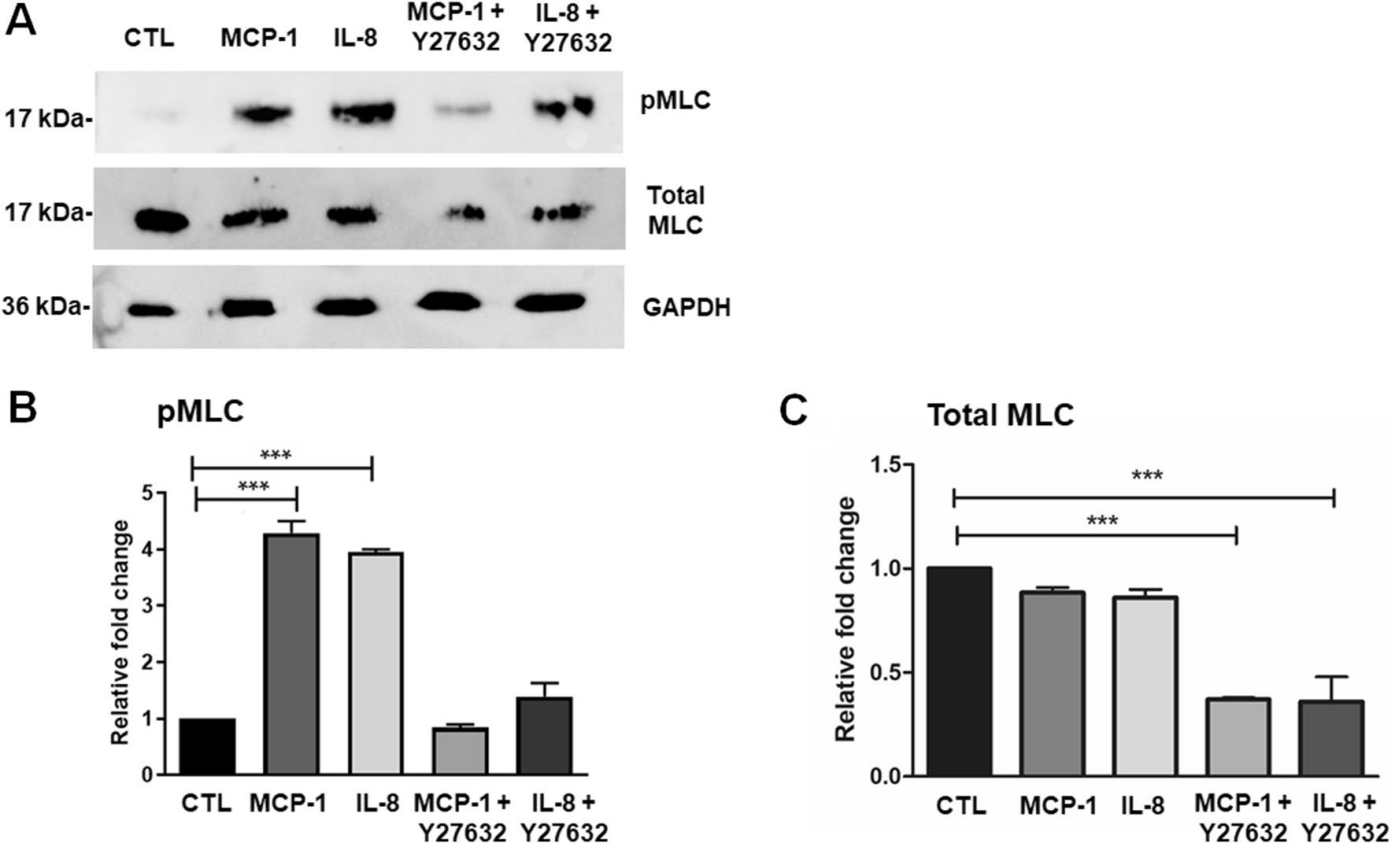
MCP-1 and IL-8 induce activation of Rho-associated protein kinase in primary cultured TM cells we generated. (**A**) Serum-starved TM cells were treated with MCP-1 (100 ng/ml) or IL-8 (100 ng/ml) alone (for 2 h) or in the presence of Rho-kinase inhibitor-Y27632 (10 µM for 30 min pretreatment) (**A**,**B**). MCP-1 and IL-8 treatment revealed significant increase in pMLC and pre-treatment with Y-27632 showed a dramatic decrease in MLC phosphorylation based on immunoblotting (**A**) and subsequent densitometric analysis (**B**). Treatment with Y-27632 also caused a significant decrease in total MLC expression (**A**,**C**). The immunoblot data were normalized to total MLC (pMLC), and to GAPDH (total MLC). Values are mean ± SEM and one-way ANOVA with Dunnett’s multiple comparison was done. ***P < 0.001, N = 3.

To ascertain the effects of MCP-1 and IL-8 on the cellular contractility, we undertook collagen gel contraction assay. After serum-starved TM cells were embedded in collagen gels and treated with different mediums for 24 and 48 h, we noted that the collagen gels were significantly contracted by MCP-1, IL-8, and TGF-β1 treatment for 24 h compared with control cells (Fig. 2A,B), which were further contracted by 48 h treatment (Fig. 2A,C). On the other hand, the effect of MCP-1 and IL-8 on collagen gel contraction was significantly suppressed in cells pre-treated with Rho kinase inhibitor, Y-27632. To determine the involvement of Rho-kinase in MCP-1 and IL-8 induced TM cell contractile activity, we also tested the effects of Y-27632 in the presence or absence of MCP-1 and IL-8 on MLC phosphorylation in TM cells. As shown in Fig. 3, treatment with MCP-1 and IL-8 increased MLC phosphorylation, which was inhibited by the pre-treatment with Y-27632 based on immunoblotting and densitometric analyses (Fig. 3A,B). TM cells treated with MCP-1 (100 ng/ml) or IL-8 (100 ng/ml) in the presence of Rho-kinase inhibitor-Y27632 showed significantly decreased levels of total MLC (Fig. 3C; uncropped images are shown in supplementary Fig S2).

**Figure 4:**
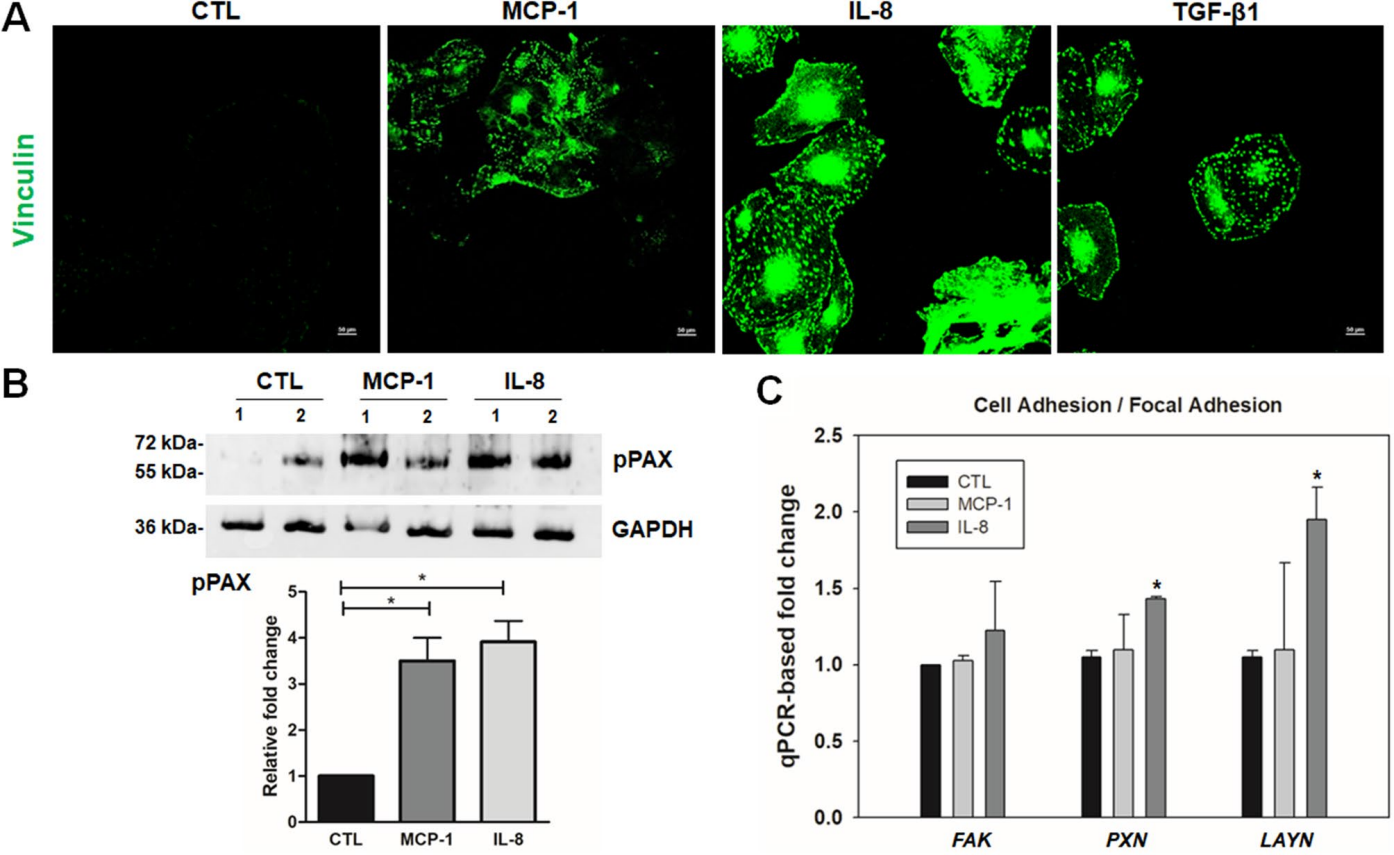
MCP-1 and IL-8 treatment induces changes in focal adhesions in human TM cells. Primary cultured TM cells we generated were cultured on 2% gelatin-coated glass cover slips and serum-starved for 24 h and treated with MCP-1 (100 ng/ml) or IL-8 (100 ng/ml) for 2 h or TGF-β1 (10 ng/ml) for 24 h before staining for focal adhesion (vinculin staining). (**A**) Representative confocal images of stained cells show an increase in focal adhesions (green) which is prominent with IL-8 treatment. TM cells treated with MCP-1 and IL-8 showed a significant increase in the level of phosphorylated Paxillin (**B**) relative to control (CTL) based on immunoblotting and densitometric analyses. The immunoblot data were normalized to GAPDH. qPCR-based confirmation of MCP-1 and IL-8 induced gene expression related to cell focal adhesion in human TM cells (*FAK, Paxillin, LAYN*) (**C**). qPCR analyses revealed a significant increase in expression of Paxillin and LAYN (in fold change) upon IL-8 treatment as compared with the controls. Full-length blots/gels are presented in Supplementary Figure S3. Values are mean ± SEM and one-way ANOVA with Dunnett’s multiple comparison was done. *P < 0.05, N = 3.

Having found that MCP-1 and IL-8 stimulate cytoskeletal reorganization and contractile activity in TM cells, we determined the effects of MCP-1 and IL-8 on the focal adhesions in TM cells. Both MCP-1 and IL-8 induced increase in the vinculin immunofluorescence staining (green), showing a robust increase especially when treated with IL-8 (Fig. 4A). Immunoblot analysis with subsequent quantification by densitometric analysis showed significant increase in the levels of phospho-paxillin with treatment of MCP-1 and IL-8 (Fig. 4B). Real-time qPCR analyses also showed a significant increase in the expression of the cell adhesion related genes (*PXN* and *LAYN*) in IL-8 treated TM cells, relative to control cells (Fig. 4C, uncropped images are shown in supplementary Fig S3).

### Increased fibrogenic activity and cell plasticity in MCP-1 and IL-8 treated TM cells

**Figure 5:**
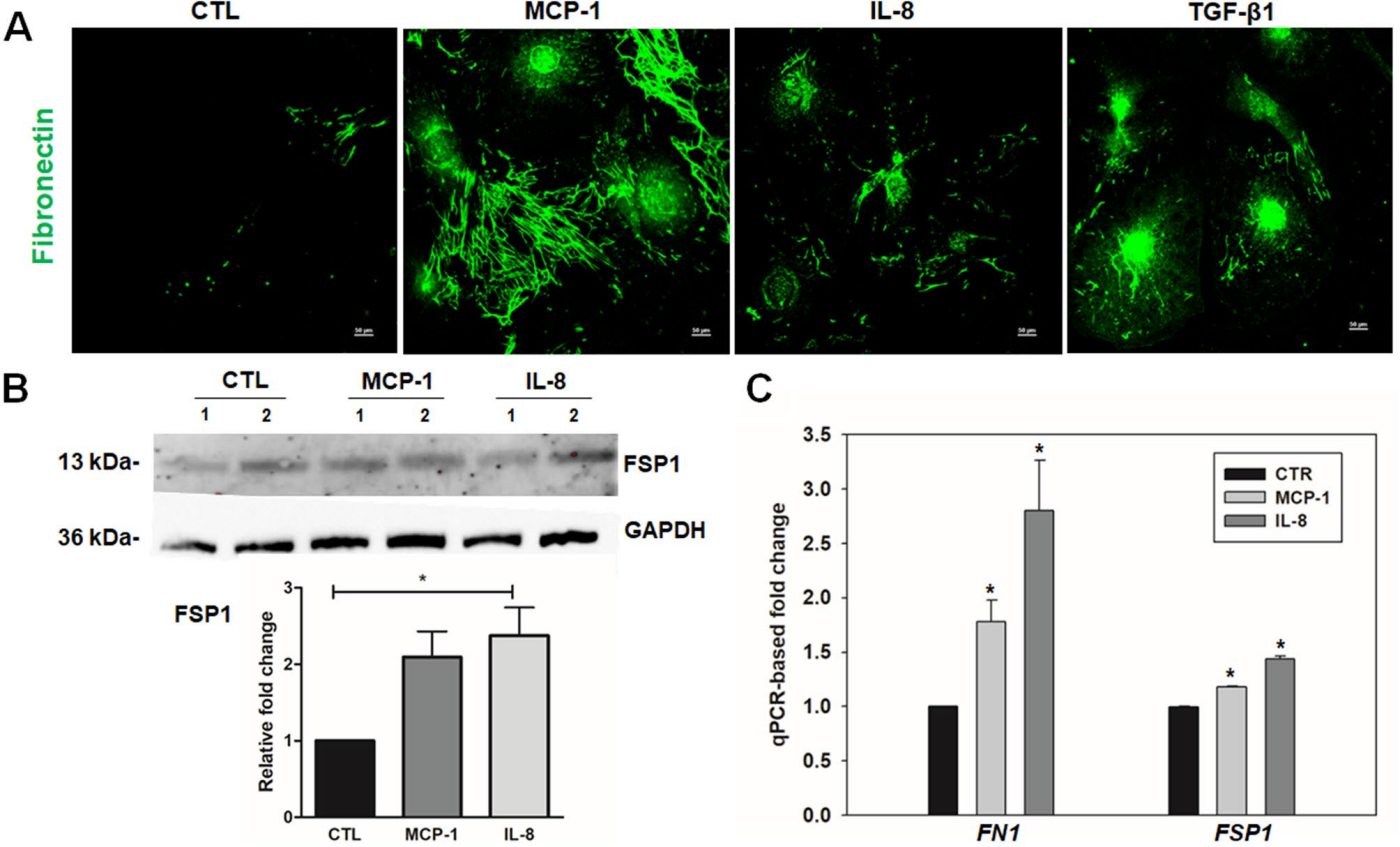
MCP-1 and IL-8 induces expression of fibrogenic and myofibroblast markers in human TM cells. Primary cultured TM cells we generated were serum starved, and treated with MCP-1 (100 ng/ml) or IL-8 (100 ng/ml) for 48 h or TGF-β1 (10 ng/ml) for 24 h before staining for fibronectin (**A**). Representative confocal images of stained cells show an increase in fibronectin (green) staining with MCP-1, IL-8, and TGF-β 1 treatment in immunoblotting and densitometric analyses (**B**). Treatment with MCP-1 and IL-8 also significantly increased the level of fibroblast specific protein (FSP)1 compared with the control group based on immunoblotting and densitometric analyses (**B**). The immunoblot data were normalized to GAPDH. (**C**) Semi-quantitative RT-PCR analysis of the same samples described in part (**A**) shows relative increase in the expression of fibrogenic genes (*FN1* and *FSP1*) in MCP-1 and IL-8 treated TM cells compared with the controls. Full-length blots/gels are presented in Supplementary Figure S4. Values are mean ± SEM and one-way ANOVA with Dunnett’s multiple comparison was done. *P < 0.05, N = 3.

In addition to addressing the regulation of TM cell contractile and adhesive characteristics by MCP-1 and IL-8, we asked whether MCP-1 and IL-8 augment fibrogenic response in TM cells. Serum-starved TM cells treated with MCP-1 (100 ng/ml) or IL-8 (100 ng/ml) for 48 h resulted in an increase in the fibronectin immunofluorescence (green) (Fig. 5A). There is an overwhelming evidence for the involvement of MCP-1 and IL-8 in the process of EMT^30,34^. Therefore, to determine the effect of MCP-1 and IL-8 in the TM cell plasticity, we tested the expression of fibroblast specific protein 1 (FSP1), also called S100A4 which is considered a marker of fibroblasts undergoing tissue remodeling and is used to identify fibroblasts derived from EMT^35^. Immunoblot and densitometric analyses showed IL-8 induced a significant increase of the expression of FSP1 (Fig. 5B, uncropped images are shown in supplementary Fig S4). Real-time qPCR analyses also showed significant increase of FN1 and FSP1 in MCP-1 and IL-8 treated TM cells, relative to control cells (Fig. 5C). Activation of EMT during wound healing exemplify individual cell migration^36^, and TGF-β1 is known to mediate the MCP-1/CCR2 induced EMT and extracellular matrix (ECM) synthesis^34^. In an effort to elucidate the effect of MCP-1 and IL-8 on cell migration in the presence or absence of TGF-β1, migration of TM cells was analyzed using the in vitro scratch assay, also known as the wound healing assay. When scratched TM cells incubated with various agents, MCP-1 and IL-8 treated TM cells showed significantly increased migration activities relative to the control cells at 24 h after scratching, and this effect was further enhanced with co-treatment with TGF-β1 (Supplementary Fig S5).

### Virus-induced changes in TM cells

**Figure 6:**
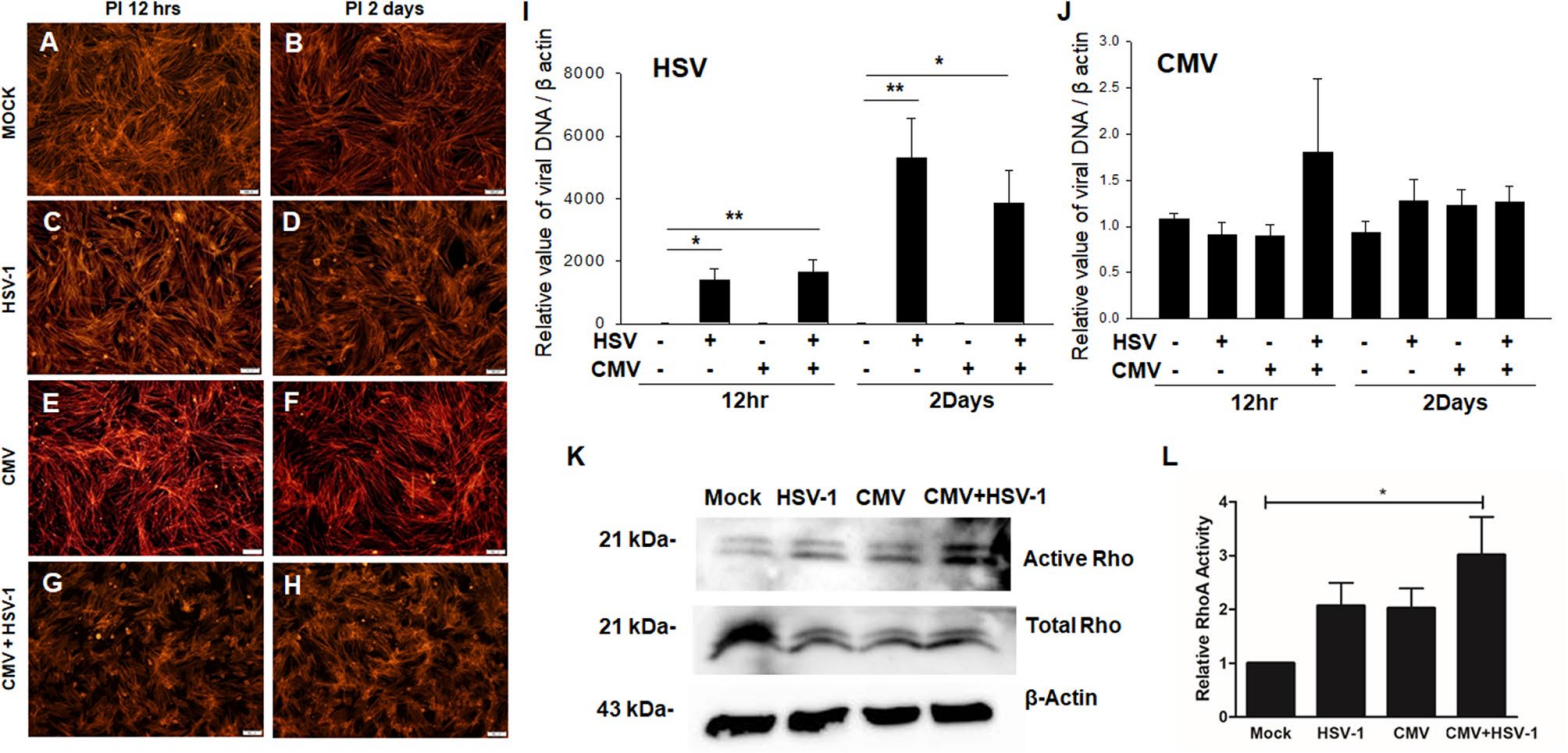
HSV-1 and CMV infection-induced changes in the human TM cell actin cytoskeletal organization, and accumulation of viral DNA at 12 h and 2 days post-infection. Primary TM cells obtained from ScienCell Research Labs were infected with HSV-1 or CMV alone, or with HSV-1 and CMV at a multiplicity of infection 1, and observed at 12 h (**A**,**C**,**E**,**G**) and 2 days post-infection (PI) (**B**,**D**,**F**, **H**). Stress fibers were stained with a Rhodamine Phalloidin (red signals) (**A**–**H**). Increased contraction of F-actin was observed after HSV-1 infection and CMV infection (**C**–**F**) when compared with mock infection (**A**,**B**). Moreover, the co-infection of HSV-1 and CMV also caused increase in F-actin formation (**G**,**H**). Cells were harvested and viral DNA was extracted from cells and processed for qPCR analysis of viral DNA accumulation using HSV-1 DNA polymerase and UL26 primer for HSV-1 (**I**) and CMV (**J**), respectively. Real-time PCR with β-actin primers were performed to serve as an internal control for input DNA. Data are the averages of three independent DNA samples from the infected cells. Activation of RhoA activity in TM cells at 2 days PI was significant after co-infection of CMV and HSV-1 in immunoblotting (**K**) and densitometric analyses (**L**). The immunoblot data were normalized to total Rho. Blots were cut prior to hybridization with antibodies. Data are shown as means ± SEM, N = 3. *P < 0.05 and **P < 0.01 vs. transcripts from the mock infection within 12 h and 2 day PI using one way ANOVA with Dunnett’s multiple comparison.

Having recognized that MCP-1 and IL-8 induce TM cell contractibility, fibrogenic activity, and cell plasticity, we tried to gain insights into the possible involvement of MCP-1 and IL-8 in the pathophysiology of virus-induced hypertensive anterior uveitis. To address this issue, we first investigated the HSV-1 and CMV infection-induced changes in actin cytoskeletal integrity and expression of cytokine/chemokine in human TM cells. The viral DNA accumulation of HSV-1 and CMV are shown in Fig. 6I,J. To investigate the actin cytoskeletal changes induced by viral infection, serum-starved TM cells were infected with HSV-1 or CMV alone or together at a MOI 1. When observed at 12 h and 2 days post-infection (PI), the HSV-1 and CMV infected TM cell exhibited a significant increase in formation of actin stress fibers compared with mock-infected TM cells (Fig. 6A–H). A pull-down assay for the active form of RhoA showed that the HSV-1 and CMV infection in TM cells induced significant activation of RhoA, which was most evident in HSV-1 and CMV co-infected TM cells in densitometric analyses at 2 days PI (Fig. 6K,L, uncropped images are shown in supplementary Fig S6 and high magnification images along with relative fluorescence density are shown in supplementary Fig S7).

**Figure 7:**
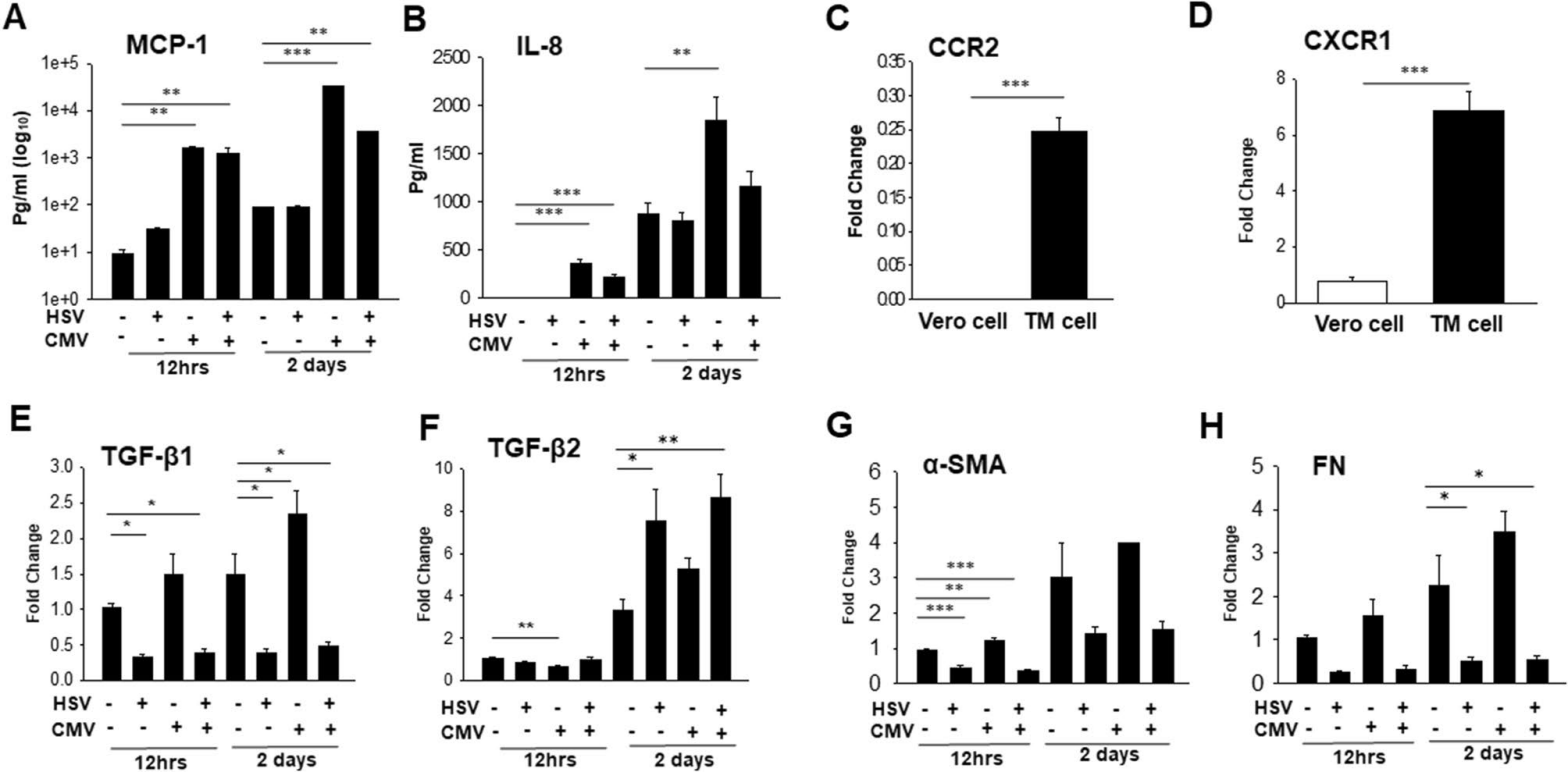
Effects of HSV-1, CMV and their co-infection on the expression of MCP-1 and IL-8 in human trabecular meshwork (TM) cells. Primary TM cells from obtained ScienCell Research Labs were infected at a multiplicity of infection of 1 and observed at 12 h and 2 days post-infection (PI). Supernatants were collected and the levels of MCP-1 (**A**) and IL-8 (**B**) were determined by ELISA assay. Expression of CCR2 (**C**) and CXCR1 (**D**), which are the receptors of MCP-1 and IL-8, respectively, are compared in the Vero cells and human TM cells using real-time qPCR analyses. The mRNA expression levels of transforming growth factor (TGF)-β1 (**E**), TGF-β2 (**F**), α-SMA (**G**), and FN (fibronectin) (**H**) are shown. HSV-1 and CMV infection induced significantly increased expression of MCP-1 and IL-8 in TM cells, showing significant and robust increase of MCP-1 and IL-8 upon CMV infection at 12 h and 2 days PI. Also, the expressions of CCR2 and CXCR1, which are the receptors of MCP-1 and IL-8, respectively, were significantly increased in TM cells, relative to Vero cells, the cell line isolated from kidney epithelial cell. (***P < 0.001 vs. transcripts from the Vero cells using independent *t* test; *P < 0.05 and **P < 0.01 vs. transcripts from the mock infection within 12 h and 2 days PI using one way ANOVA with Dunnett’s multiple comparison, N = 3).

Having found that HSV-1 and CMV infection induces actin cytoskeletal contraction in TM cells, we asked whether the HSV-1 and CMV infection induces the expression of MCP-1 and IL-8 as well as the cytokines and fibrogenic proteins associated with glaucoma in TM cells (Fig. 7). ELISA assay showed that CMV infection and co-infection of HSV-1 and CMV induced significantly increased expression of MCP-1 and IL-8 in TM cells. This response was found to be robust upon CMV infection at 2 days PI (Fig. 7A,B). In addition, the expressions of CCR2 and CXCR1, which are the receptors of MCP-1 and IL-8, respectively, were significantly increased in TM cells, relative to Vero cells, the cell line isolated from kidney epithelial cell (Fig. 7C,D).

Transforming growth factor-β (TGF-β), an upstream molecule that increases resistance of the outflow pathway, is sufficiently present in the AH to serve a purpose in normal ocular physiology, and is found in increasing amounts in the AH of patients with POAG^37^. In our previous study^38^, we had obtained evidence that CMV infection in human TM cells inducing expression of TGF-β1, which has been shown to increase the resistance of the outflow pathway^19^. We confirmed that infection of CMV in TM cells significantly enhanced the expression of TGF-β1 at 2 days PI (Fig. 7E). In contrast, HSV-1 infection significantly reduced the expression of TGF-β1, α-SMA, and fibronectin, whereas it increased the expression of TGFβ-2 in TM cells (Fig. 7E–H).

Since virus infection in TM cells induced robust elevation of the inflammatory chemokines, MCP-1 and IL-8 as well as modest elevation of TGF-β, we then evaluated the possible stimulatory or inhibitory effect between MCP-1, IL-8 and TGF-β1. When serum-starved TM cells were treated with MCP-1 (100 ng/ml) or IL-8 (100 ng/ml) in the presence or absence of TGF-β1 (15 ng/ml), we found that TM cells treated with TGF-β1 showed significantly decreased expression of IL-8 in TM cells (Supplementary Fig S8F) relative to control cells. However, MCP-1 treated TM cells showed significantly elevated expression of MCP-1 and IL-8 (Supplementary Fig S8B,C), and similarly, IL-8 treated TM cells also showed increased expression of IL-8 (Supplementary Fig S8F).

## Discussions

**Figure 8:**
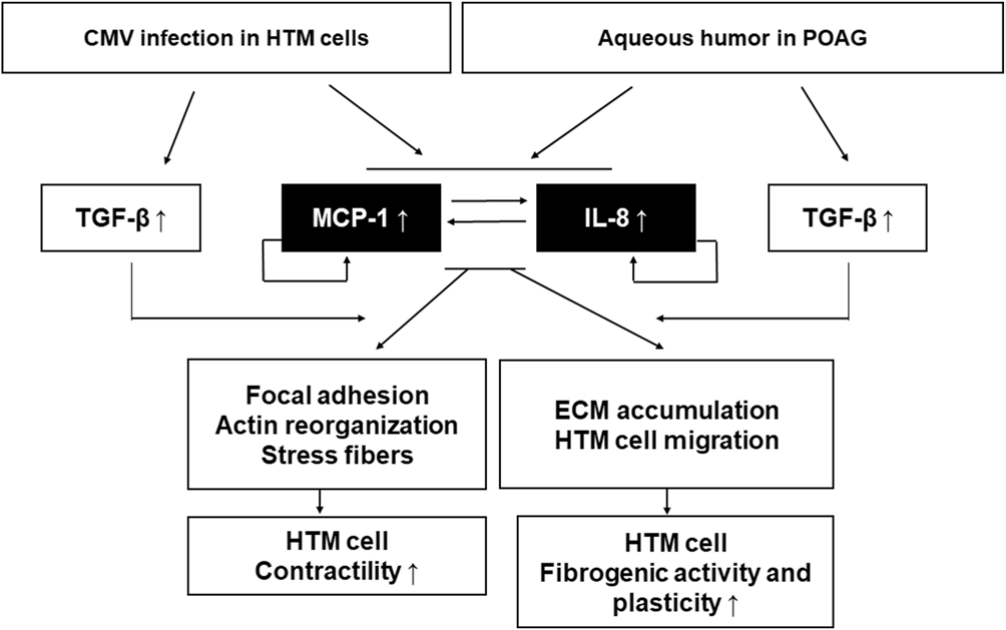
Schematic illustration of the role and regulatory effects of MCP-1 and IL-8 in human TM cells. CMV or HSV-1 infection in human TM cells dramatically increases the expression of inflammatory chemokines MCP-1 and IL-8 as well as TGF-β, which is also increased in the aqueous humor of primary open angle glaucoma (POAG). TM cells express the receptors of MCP-1 and IL-8 (CCR2 and CXCR1, respectively), and MCP-1 and IL-8 are augmented in an autocrine manner, and exhibited reciprocal regulatory effects on each other, while TGF-β inhibited the expression of MCP-1 and IL-8. Short-term treatment with MCP-1 and IL-8 increases the focal adhesion, actin reorganization, and stress fibers via Rho-A pathway, which collectively increases TM cell contractibility. Long-term treatment with MCP-1 and IL-8 enhanced EMT markers, ECM accumulation and TM cell migration, indicating increased fibrogenic activity and cell plasticity. This short term and long-term effects of inflammatory chemokines might contribute to the increased resistance to the aqueous humor outflow facility in hypertensive anterior uveitis and POAG.

Toward the goal of understanding the role of the inflammatory chemokines, MCP-1, and IL-8 in the pathophysiology of POAG, and hypertensive anterior uveitis, we investigated their effects on the regulation of TM cell contractile activity, and the cellular events relevant to the AH outflow dynamics. Short-term treatment with MCP-1 and IL-8 increased the focal adhesions formation, actin reorganization, and actin stress fibers via Rho-A activation, which collectively increased TM cell contractibility. Long-term treatment with MCP-1 and IL-8 enhanced the TM cell plasticity, ECM accumulation, cell migration, thereby increasing fibrogenic activity. In the literature review for the last decade, the levels of MCP-1 and IL-8 in the AH of patients with POAG and hypertensive anterior uveitis such as PSS and FUS was reportedly elevated than controls (Supplementary Table S1). In our study, CMV infection in human TM cells, which are important causative factors in hypertensive anterior uveitis, dramatically increased the expression of inflammatory chemokines MCP-1 and IL-8. Collectively, these findings suggest that the short term and long-term effects of MCP-1 and IL-8 might contribute to the increased resistance to outflow facility in patients with hypertensive anterior uveitis and those with POAG (Fig. 8).

Human TM cells play a key role in homeostasis of IOP, by regulating flow resistance in the AH outflow pathway^31,39,40^. To regulate the functions of the cells and extracellular matrix, TM cells secrete abundant factors, including various cytokines, chemokines, and matrix metalloproteinase. Non-glaucomatous TM cells secrete significant quantities of the chemokines IL-8, MCP-1, and CXCL6 in absence of any stimulation^32^. In a former study by Tsuboi et al.^41^, it has been shown that CCR2, a receptor of MCP-1, is highly expressed in porcine TM cells. In line with the previous finding, we demonstrated that CCR2, as well as CXCR1, the specific receptor of IL-8, are highly expressed in human TM cells, compared with the control Vero cells (Fig. 7). Not only the constitutive secretion of MCP-1 and IL-8 by human TM cells but also the expression of their receptors strongly suggests that these inflammatory chemokines play a physiologic role in the maintenance of human aqueous outflow.

Clinical clues suggesting virus etiology in the hypertensive anterior uveitis include anterior uveitis with granulomatous keratic precipitates manifesting as PSS-like uveitis or FUS-like uveitis^42^, and the most commonly implicated viruses are HSV, CMV, and VZV^20^. Especially, CMV is increasingly recognized as an important cause of hypertensive anterior uveitis in immunocompetent individuals^20,21,42^. In association with the clinical findings, we found that the HSV-1 and CMV infection caused increased cytoskeletal contraction associated with significant activation of Rho-A (Fig. 6 K,L). The relatively low total Rho protein level in the virus-infected TM cells compared with Mock-infected TM cells could have been affected by cell death caused by viral infection. The cells might have been metabolically compromised since some of the virus-infected cells are dying. Further studies regarding the direct involvement of MCP-1 and IL-8 in virus-induced cytoskeletal contraction are necessary.

Inflammatory cytokines or chemokines play an important role in the process of HSV-1 and CMV infection. At the early stage of HSV-1 infection, MCP-1 and IL-8 are induced for recruitment of leukocyte to the viral replication site^43^. Intrinsically, CMV has immunomodulatory properties and immune evading strategies^44^. Monocyte, the immune effector cell targeted by CMV works as the cell reservoir for latency and dissemination, which renders increased expression of MCP-1 and IL-8 in CMV infected cells^44^. In association with the above described studies, we found that the expression of MCP-1 and IL-8 was significantly increased with viral infection in human TM cells, especially with CMV infection, showing a robust increase in the expression of MCP-1 and IL-8, respectively. Although HSV-1 induced increased expression of MCP-1 at 12 h PI (Fig. 7A), it was not significant as the effect of CMV infection. Moreover, HSV-1 infection decreased expression of TGF-β1 and increased expression of TGF-β2, whereas CMV infection induced expression of MCP-1, IL-8 and TGF-β1 in TM cells (Fig. 7A,B,E,F). In this regard, CMV and HSV-1 infection seems to exhibit differential mechanisms associated with ocular hypertension in hypertensive anterior uveitis.

In our study, MCP-1 and IL-8 significantly induced actin stress fibers and focal adhesions in association with increased phosphorylation of MLC and paxillin in TM cells, which demonstrates that these chemokines play a role in regulation of cellular contractility and adhesive interactions, thereby potentially regulating AH outflow and IOP. As a chemokine, MCP-1 is a potent chemoattractant for monocytes, and IL-8 for neutrophil, triggering the firm adhesion of the inflammatory cells to vascular endothelium under flow conditions^45^. Chemokines exert their effect, by binding to G-protein coupled receptors on the cell surfaces of target cells. For cell motility through actin-polymerization, chemokine receptor also activates Rho-family proteins. Janani et al.^28^ reported that MCP-1 induced increased mobility of vascular smooth muscle cells via actin polymerization through phosphorylation of contractin. Stimulation with IL-8 not only led to actin polymerization in neutrophil^46^, but also the activation of endothelial cells through the Rho signaling pathway^29^. IL-8 was also found to mediate the contraction in human airway smooth muscle cells through CXCR^47^. In association with the former findings, we showed that MCP-1 and IL-8 increase the cellular contractile activity via activation of the Rho A signaling pathway, which was inhibited by the Rho-kinase inhibitor, Y-27632. Furthermore, induction of focal adhesions was more apparent by the stimulation with IL-8 (Fig. 4). This is in line with the former finding that IL-8 induced cell migratory activities are dependent on the phosphorylation of focal adhesion proteins and focal adhesions distribution^48^. In this regards, it is proposed that the abrupt increase of MCP-1 and IL-8 in pathologic conditions may exert direct influence on the TM cells contractile activity via their abundantly expressed receptors, which possibly results in IOP elevation.

Regarding to the effect of MCP-1 on the AH outflow pathway, Alvarado et al.^49^ found that substantial numbers of macrophages are recruited after selective laser trabeculoplasty which is the technique used with the purpose of lowering IOP. They suggested that macrophages which were recruited by the release of chemokines such as MCP-1 and IL-8, play a role in the increased permeability in Schlemm canal endothelial cells. In addition, in a study of Tsuboi et al.^41^. MCP-1 itself has been found to increase the permeability of Schlemm’s canal endothelial cells. Considering that MCP-1 is constitutively secreted by human TM cells, MCP-1 might have a regulatory role in normal ocular physiology. In this regard, there seems to be differential effects of MCP-1 according to the cell types in the outflow pathway.

Intriguingly, we found that MCP-1 and IL-8 augmenting the fibrogenic response and migration activities in human TM cells. There is substantial evidence suggesting that MCP-1 and IL-8 are involved not only in cell migration but also in EMT phenomenon^29,30,50^. In association with our study, Kupper et al.^50^ showed that the autocrine MCP-1/CCR2 signaling increased cell proliferation and migration. Also, via the Rac1/RhoA-p38MAPK signaling pathway, IL-8 was found to promote endothelial cell migratory activity, and thereby promoting tumor angiogenesis^29^. Especially, MCP-1/CCR2 and IL-8/CXCR signaling is known to induce EMT in various cancer cell lines, which are considered to be potential targets of tumor progression and metastasis^30,34^. Furthermore, MCP-1 induced EMT in peritoneal mesenchymal cells was partly mediated by TGF-β1^34^, which is known to enhance cell plasticity and fibrogenic activity via RhoA pathway^39^. In normal AH, TGF-β is sufficiently present with a purpose to serve normal ocular physiology, and is found to be upregulated in POAG eyes. In this study, CMV infection significantly increased the expression of TGF-β1 in TM cells. The increased migration activity in MCP-1 and IL-8 treated TM cells was significantly enhanced with co-treatment with TGF-β1 (Supplementary Fig S5). These findings suggest that TGF-β could augment the fibrogenic activity and cell plasticity caused by elevated levels of MCP-1 and IL-8 in pathologic conditions.

Regarding the interaction between chemokines, we found that TGF-β inhibited the expression of MCP-1 and IL-8, whereas MCP-1 and IL-8 are augmented in an autocrine manner and exhibited reciprocal regulatory effects on each other. There seems to be a regulatory loop between autocrine MCP-1/CCR2 and IL-8/CXCR secretion and TGF-β1. Also, in glomerular cells, an intrinsic feedback loop has been found: increased MCP-1 levels induce TGF-β, whereas increased TGF-β exerts a negative feedback on MCP-1^51^. Considering that TGF-β, MCP-1 and IL-8 are up-regulated in glaucomatous eyes, further investigations are needed to target for the reciprocal regulatory interaction between TGF-β and inflammatory chemokines.

In summary, we found that MCP-1 and IL-8 play a significant role in the AH outflow pathway by increasing the actin stress fibers in association with increased phosphorylation of MLC, focal adhesions, and fibrogenic activity. We also showed that especially CMV infection which is an important cause of hypertensive anterior uveitis, induced robust expression of MCP-1 and IL-8 in human TM cells. Our study demonstrates a role for MCP-1 and IL-8 in regulation of cellular contractile and adhesive interactions of TM cells which are recognized to influence AH outflow and IOP in hypertensive anterior uveitis and POAG.

## Methods

### Chemicals

MCP-1 (Cat. no.279-MC-010; R&D systems, Minneapolis MN), IL-8 (Cat. No. 208-IL-010; R&D systems), recombinant TGF-β1 (Cat. No. 240-B-010; R&D system), and Y-27632 (Cat. No. 1254; Tocris Bioscience, Bristol, UK) were purchased from the respective commercial sources.

### Cells

Primary human TM cell cultures were derived from TM tissue isolated from freshly obtained donor corneal rings (from three different human donors aged 27, 44, and 60 years) used for corneal transplantation at the Duke Ophthalmology clinical service upon informed consent. The TM culture was done within 48 h of death, previously described^40^. The protocols involving the use of human tissue were approved under the Duke University Institutional Review Board (Pro00093311) and were consistent with the tenets of the Declaration of Helsinki. The extracted TM tissue was chopped into small pieces in 100% fetal bovine serum (FBS), and then were placed under a glass coverslip in six-well plastic culture plates and cultured in Dulbecco’s modified Eagle medium (DMEM) containing 20% FBS and penicillin (100 U/500 ml)-streptomycin (100 μg/500 ml)-glutamine (4 mM) and incubated in a humidified atmosphere of 5% CO_2_ at 37 °C. Cells derived from the TM tissue were passaged and used for the experiment at the passage 3–6. For experiments regarding TM cell contraction assay (Fig. 2), viral infection (Figs. 6, 7) and wound healing assay (Supplementary Fig S5), primary TM cells obtained from ScienCell Research Labs (Carlsbad, CA, USA) were used and cultured to 100% confluence in Trabecular Meshwork Cell Medium (catalog No. 6591; ScienCell Research Labs)^38,52^. Characterization of the primary TM cells obtained from ScienCell is shown in Supplementary Fig S9 and S10. All cells were serum-starved for 24 h prior to treatment with various agents unless mentioned otherwise.

### Viral infection

For some experiments, confluent TM cells were mock-infected or infected with HSV-1 or CMV alone, or with HSV-1 and CMV at a multiplicity of infection (MOI) of 1. HSV-1 clinical strain NCCP no. 43002 was provided by the Korea Centers for Disease Control and Prevention (Osong, Republic of Korea). Vero cells were used to propagate virus progenies, and standard plaque titrations were performed on Vero cells^53^. Human CMV strain AD169 was propagated using HFF (human foreskin fibroblast) cells, and virus stocks were titrated using a 50% tissue culture infectious dose (TCID_50_) assay on HFF cells, using the method of Reed and Muench^54^. After viruses were adsorbed for 2 h on confluent TM cells, the infected cells were washed once with 1 × phosphate-buffered saline (PBS) and maintenance medium was applied. At 12 h and 2 days PI, analyses of HSV-1, CMV, and HSV-1 and CMV co-infected TM cells were performed^55^. All procedures adhered to the tenets of the Declaration of Helsinki.

### Viral DNA replication assays

From the TM cells mock-infected or infected with HSV-1 or CMV alone, or with HSV-1 and CMV, viral DNA was harvested from the cells in 12-well plates, and isolated using the Qiagen column (QIAmp DNA Mini Kit; Qiagen, Hilden, Germany) at 12 h and 2 days PI. The viruses were infected at a multiplicity of infection (MOI) of 1 based on published literatures^38,56^. The replicated viral DNA was quantified using real-time polymerase chain reaction (PCR) using HSV-1 DNA polymerase primers for HSV-1^57^, or UL26 primers for CMV, as described previously^58^. As an internal control for input DNA, real-time PCR with β-actin primers was also performed. The primer sequences are listed in Supplementary Table S2.

### Immunocytochemistry

Human TM cells were grown on gelatin (2%)-coated glass coverslips until attainment of 70–80% confluency. TM cells treated with MCP-1 (100 ng/ml, 2 h or 48 h)^59^, IL-8 (100 ng/ml, 2 h or 48 h)^47^, or with TGF-β1 (10 ng/ml, 24 h) and virus-infected or mock-infected cells were washed with PBS and fixed with 4% formaldehyde for 15 min. After permeabilization, the cells were blocked and stained for F-actin with Tetramethylrhodamine (TRITC)-phalloidin (C.No.P1951; Sigma-Aldrich Corp., St. Louis, MO, USA), vinculin with mouse monoclonal anti-vinculin antibody (C.No.V9131; Sigma-Aldrich Corp.), and fibronectin with rabbit polyclonal antibody (obtained from Herald Erickson, Duke University) in 10% FBS/PBS (primary antibodies were omitted as negative controls; data not shown), and appropriate secondary antibodies conjugated with Alexa Fluorophores were applied for 2 h at room temperature as described previously^60^. Cell nuclei were counterstained with 4′,6-diamidino-2-phenylindole (DAPI) (Vectashield with DAPI, Vector Laboratories). Coverslips were mounted onto glass slides using Shandon Immu-Mount (Thermo-Fisher Scientific), then imaged using a Nikon Eclipse 90i confocal laser-scanning microscope. Intensity of fluorescence was measured in a relative arbitrary unit under the same conditions and settings for each sample using imageJ software (v.1.53).

### Immunoblotting

From serum-starved cultures of TM cells treated with MCP-1 (100 ng/ml) or IL-8 (100 mg/ml) for 2 h or 48 h, total protein cell lysates were prepared and homogenized at 4 °C in hypotonic 10 mM Tris buffer, pH 7.4, containing 0.2 mM MgCl_2_, 5 mM N-ethymaleimide, 2.0 mM Na_3_VO_4_, 10 mM NaF, 60 uM phenylmethy sulfonyl fluoride, 0.4 mM iodoacetamide, and protease and phosphatase inhibitor cocktail (one tablet each/10 ml buffer), using a probe sonicator. From cell lysate, protein concentration was determined using protein assay reagent (Pierce 660 nm Protein Assay Reagent, ThermoFisher Scientific). Samples containing equal amounts of protein were mixed with Laemmli buffer and separated by SDS-PAGE (5–12% acrylamide), followed by transfer of resolved proteins to nitrocellulose membranes. After blocking for 2 h at room temperature in Tris-buffered saline containing 0.1% Tween 2 and 5% (wt/vol) non-fat dry milk, nitrocellulose membranes were probed with primary antibodies (at 1:1000 dilution) directed against phospho-MLC (polyclonal, C. No.3674; Cell Signaling Technology); MLC (polyclonal, C. No. 3672; Cell Signaling Technology), phospho-MYPT1 (polyclonal, C. No. ABS45; Millipore, Billerica, MA, ISA), phospho-paxillin (polyclonal, C. No. 2541, Cell Signaling Technology, Danvers, MA, USA), fibronectin (1:15,000 dilution; polyclonal C. No. ab23750; Abcam, Cambridge, MA, USA), FSP1/S100A4 (polyclonal C. No. 07-2274, Sigma-Aldrich), and GAPDH (mouse monoclonal antibody at 1:10,000 dilution; C. No. 60004-1g, Proteintech Group, Inc. Rosemont, IL, USA). After washing off the primary antibodies, membranes were incubated with horseradish peroxidase-conjugated secondary antibodies (1:5000 dilution) for 2 h at room temperature and immunopositive protein bands were detected with an enhanced chemiluminescence. Urea-glycerol gels were used for phospho-MLC immunoblots, as we described previously^60^. Densitometric analysis of immunoblots was performed using ImageJ software (http://imagej.nih.gov/il/; provided in the public domain by the National Institutes of Health, Bethesda, MD, USA). Data were normalized relative to the specified loading controls.

### RNA extraction and real-time PCR

Total RNA was extracted using RNeasy Mini Kit; Qiagen, Valencia, CA), and for first-strand cDNA synthesis, a cDNA synthesis kit (PrimeScript RT reagent Kit, Takara, Japan) was used. The relative expression levels of mRNA were determined using a Roche Diagnostics LightCycler 2.0 Real-Time PCR System (Roche GmbH, Mannheim, Germany) according to the manufacturer’s instructions. Real-time qPCR was done on the resultant reverse transcriptase-derived single stranded cDNA using sequence-specific forward and reverse oligonucleotide primers for the target genes (Supplementary Table S2), as previously described^38^. Reactions for each sample were run in triplicate, cycle thresholds were normalized to β-actin expression, and comparative quantitation was performed (LightCycler software, version 4.1, Roche). Only individual PCR samples with single-peak dissociation curves were selected for data analysis.

### Rho activation assay

The TM cells were cultured on 10 cm diameter dishes. After the cells were grown to 100% confluence, the TM cells were infected with HSV-1 or CMV alone, or with HSV-1 and CMV together at a MOI of 1. At 2 days PI, RhoA activation was evaluated using a Rho pull-down activation assay using a commercial kit (Cat no. BK036, Cytoskeleton, Denver, CO, USA) in mock-infected and infected TM cells. Briefly, Rhotekin-Rho binding domain beads were used to pulldown the active form of RhoA. The beads were washed three times with lysis buffer, and the bound GTP-Rho was detected by immunoblotting analyses with mouse anti-human monoclonal IgM RhoA antibody (Cat no. ARH04; Cytoskeleton, Denver, CO; 1:500), followed by goat anti-mouse horseradish peroxidase-conjugated secondary antibody (1:2000). Active RhoA (GTP binding form)-immunoreactive bands were visualized with enhanced chemoluminiscence.

### Collagen contraction assay

After treatment with trypsin–EDTA for 5 min, the TM cells were collected and re-suspended in serum-free medium at 3 × 10^6^ cells/ml total 9.54 ml of a collagen type I (3 mg/ml; Cat. No. A1048301, Gibco), 2.46 ml of 5X PBS, and 340 μl of neutralization solution were mixed in an ice bath to make collagen gel working solution. The collagen lattice was prepared by mixing the cell suspension and the collagen working solution (2:8). Then, 0.5 ml of the cell-collagen mixture was transferred each well in a 24-well plate, and incubated for 1 h at 37 °C. After collagen polymerization, 1 ml of DMEM, with or without Y-27632 (10 μM) was added on the top of the collagen gels. After 30 min of pre-treatment, 0.5 ml of DMEM containing MCP-1 (100 ng/ml), IL-8 (100 ng/ml), or TGF-β1 (15 ng/ml) were supplemented. The gels were carefully detached from the bottom of the wells with a 10 µL pipette tip. After 24 and 48 h, the areas of the collagen gels were measured and analyzed using imageJ software. The area of the gels containing untreated TM cells was used for normalization, and relative changes were shown as a bar chart.

### Measurements of wound healing (cell motility)

The TM cells were grown to confluence in 6-well plates and incubated with serum-free media for 24 h. The medium was removed and replaced with MCP-1 (100 ng/ml) or IL-8 (100 ng/ml) in the presence or absence of TGF-β1 (15 ng/ml). Serum-free medium was used as negative control and TGF-β1 was used as positive control. Subsequently, a wound was created manually scraping the cell monolayer with a 200 µl pipet tip. Immediately following scraping, the initial wound was photographed digitally using fluorescence microscopy (IX83, Olympus, Corporation, Tokyo, Japan). The images of the progress of cells moving into the wound area were acquired after incubation at 37 °C for 4 and 24 h and quantitatively analyzed using ImageJ software. The wound area of untreated cells at 0 h was set at 100%, and the mean percentage of the wound areas of TM cells with various treatment were calculated.

### ELISA for MCP-1 and IL-8

Levels of secreted MCP-1 and IL-8 were measured by determining their concentration in conditioned medium using a commercially available sandwich enzyme-linked immunosorbent assay (ELISA, Human CCL2/MCP-1 and Human IL-8/CXCL8 Immunoassay, R&D systems, Minneapolis, MN, USA), as previously described^38^. Conditioned medium was harvested on 12 h and 2 days PI, cleared by centrifugation, and stored at − 70 °C. Conditioned medium was acid-activated and directly assayed using an ELISA plate reader at 450 nm, according to the manufacturer’s instructions. Protein concentrations were calculated from a standard curve with two-fold serial dilutions and a highest standard of 2000 pg/ml.

### Statistical analyses

Experiments were performed in triplicate and representative results are shown. All data were expressed as mean ± standard error of the mean (SEM) values based upon analysis of at least three independent samples, until otherwise mentioned. One way analysis of variance (ANOVA) with Dunnett’s multiple comparison was done for comparing within groups and Student’s *t* test was used for all other statistical comparison between two groups. A value of *P* < 0.05 was considered statistically significant.

## Supplementary Information

Supplementary Information 1.
