# Supplementary material for: Role of MCP-1 and IL-8 in viral anterior uveitis, and contractility and fibrogenic activity of trabecular meshwork cells
Source: Sci Rep. 2021 Jul 22;11:14950. doi: 10.1038/s41598-021-94391-2 (PMC8298573; doi:10.1038/s41598-021-94391-2)
Supplement: Supplementary file 1 — Supplementary Information 1. [file 41598_2021_94391_MOESM1_ESM.docx]

***Scientific Reports***

**Role of MCP-1 and IL-8 in Viral Anterior Uveitis, and Contractility and Fibrogenic Activity of Trabecular Meshwork Cells**

Jiyoung Lee^1^, Jin A Choi^1,2¶^, Hyun-hee Ju^1^, Ju-Eun Kim^3^,

Soon-young Paik^3^, Ponugoti Vasantha Rao^2,4^

**Word count: 5,708**

1. Department of Ophthalmology, College of Medicine, St. Vincent’s Hospital, The Catholic University of Korea, Seoul, Republic of Korea

2. Department of Ophthalmology, Duke University School of Medicine, Durham, North Carolina, United States

3. Department of Microbiology, College of Medicine, The Catholic University of Korea, Seoul, Republic of Korea

4. Department of Pharmacology and Cancer Biology, Duke University School of Medicine, Durham, North Carolina, United States

**¶Correspondence: Jin A Choi, MD, PhD**

Department of Ophthalmology and Visual Science, College of Medicine, St. Vincent’s Hospital, The Catholic University of Korea, Banpo-daero 222, Seocho-gu, Seoul 137-701, Republic of Korea

**Tel: 82-031-881-8971, Email: jinah616@catholic.ac.kr**

**Table S1. Elevated aqueous humor concentration of MCP-1 and IL-8 in open angle glaucoma and hypertensive anterior uveitis in the literature**

| Study / year | Patients | n | Concentration (pg/ml) | Control | n | Concentration | *p* value |
| --- | --- | --- | --- | --- | --- | --- | --- |
| Zenkel et al., 2010^7^ | PEX | 14 | MCP-1; 583.0 ± 319.0  IL-8; 25.5 ± 23.5 | Cataract | 14 | MCP-1; 536.0 ± 268.0  IL-8; 10.1 ± 6.6 | NS^*^  *p* < 0.005^†^ |
| Kuchtey et al., 2010^17^ | POAG | 29 | IL-8; 4.9 | Cataract | 30 | IL-8; 1.8 | *p* < 0.001^†^ |
| Li et al., 2012^22^ | PSS | 53 | MCP-1; CMV (+) PSS: 401.1  CMV (-) PSS: 459.9  IL-8; CMV (+) PSS; 24.0  CMV(+) PSS : 23.5 | Cataract | 23 | MCP-1; 180.9  IL-8; 3.0 | *p* =0.026 (CMV (+) PSS) ^*^  *p* = 0.004 (CMV (-) PSS) ^*^  *p* =0.054 (CMV(+) PSS) ^†^  *p* = 0.029 (CMV (-) PSS) ^†^ |
| Takai et al., 2012^10^ | POAG | 20 | IL-8; 3.2 ± 1.9 | Cataract | 21 | IL-8; 1.4 ± 1.2 | *p* =0.0004^†^ |
|  | PEXG | 23 | IL-8; 5.6 ± 3.6 |  |  |  | *p* <0.0001^†^ |
| Inoue et al., 2012^9^ | POAG (phakic) | 23 | MCP-1; 1142.5  IL-8; 15.2 | Cataract | 52 | MCP-1; 829.4  IL-8; 4.6 | *p* < 0.01^*^  *p* < 0.01^†^ |
|  | PEXG (phakic) | 26 | MCP-1; 1253.9  IL-8; 12.3 |  |  |  | *p* <0.01^*^  NS^†^ |
| Chua et al., 2012^16^ | Glaucoma | 38 | MCP-1; 295.22  IL-8; 5.58 | Cataract | 23 | MCP-1; 180.89  IL-8; 3.08 | *p* = 0.086^*^  *p* = 0.060^†^ |
| Freedman et al., 2013^8^ | POAG | 23 | MCP-1; 178 ± 33  IL-8; 39 ± 10 | Cataract | 13 | MCP-1; 40 ± 18  IL-8; 18 ± 12 | *p* < 0.05^*^  NS^†^ |
| Ohira et al., 2015^25^­­­­­­­­ | UG | 39 | MCP-1; 2791.7 ± 1927.3  IL-8; 214.5 ± 721.7 | Cataract | 68 | MCP-1; 971.3 ± 500.5  IL-8; 6.0 ± 9.2 | *p* < 0.01^*^  *p* < 0.05^†^ |
|  | POAG | 36 | MCP-1; 1293.1 ± 445.9  IL-8; 17.0 ± 23.6 |  |  |  | NS^*^  NS^†^ |
| Garweg et al., 2017^11^ | Early PEX | 33 | MCP-1; 368.1 ± 125.5  IL-8; 3.7 ± 1.4 | Cataract | 20 | MCP-1; 244.5 ± 68.2  IL-8; 3.7 ± 2.1 | *p* =0.003^*^  NS^†^ |
|  | Late PEX | 30 | MCP-1; 361.6 ± 117.1  IL-8; 6.5 ± 4.1 |  |  |  | *p* = 0.006^*^  *p* = 0.059^†^ |
| Khalef et al. 2017^12^ | POAG | 30 | IL-8; 3.2±1.9 | Cataract | 15 | IL-8; 1.5±1.2 | < 0.001^†^ |
| Kokubun, et al. 2018^14^ | POAG | 24 | MCP-1; 519.7 ± 376.8  IL-8; 33.6 ± 35.5 | Cataract | 21 | MCP-1; 309.6 ± 199.6  IL-8; 9.0 ± 10.7 | *p* = 0.0116^*^  *p* = 0.0011^†^ |
|  | NVG | 9 | MCP-1; 648.4 ± 202.8  IL-8; 65.0 ± 84.7 |  |  |  | *p* = 0.0007^*^  *p* = 0.0002^†^ |
| Pohlmann et al. 2018^23^ | PSS | 16 | MCP-1;1267  IL-8; 199.4 | Cataract | 11 | MCP-1;957.6  IL-8;56.5 | *p* = 0.1623^*^  *p* = 0.0089^†^ |
|  | FUS | 65 | MCP-1;1561  IL-8;72,4 |  |  |  | *p* = 0.0013^*^  *p* = 0.1441^†^ |
| Chono et al. 2018^13^ | POAG | 31 | MCP-1; NA (<500)  IL-8; NA (<10) | Cataract | 100 | MCP-1; NA (<500)  IL-8; NA (<10) | NS^*^  NS^†^ |
|  | PXG | 38 | MCP-1; NA (~1300)  IL-8; NA (~50) |  |  |  | *p* < 0.05^*^  *p* = 0.0000^†^ |
|  | NVG | 32 | MCP-1; NA (~2900)  IL-8; NA (~620) |  |  |  | *p* = 0.0000^*^  *p* = 0.0000^†^ |
| Bauer, et al. 2019^24^ | FUS without Glaucoma | 11 | MCP-1; 500.97 ± 226.19  IL-8; 2.46 ± 2.45 | Cataract | 24 | MCP-1; 119.94 ± 37.28  IL-8; 0.25 ± 0.12 | *p* < 0.0001^*^  *p* < 0.001^†^ |
|  | FUS with Glaucoma | 8 | MCP-1; 239.70 ± 112.41  IL-8; 5.11 ± 5.09 |  |  |  | *p* = 0.0001^*^  *p* < 0.001^†^ |
| Pantalon, et al. 2019^15^ | POAG | 24 | MCP-1; 383.59 ± 249.32  IL-8; 22.02 ±13.61 | Cataract | 16 | MCP-1; 449 ± 260.81  IL-8; 12.13 ± 3.68 | NS^*^  NA^†^ |
| Burgos-Blasco et al, 2020^6^ | POAG | 27 | MCP-1; 268.36 ± 36.38  IL-8; 642.01 ± 411.71 | Cataract | 29 | MCP-1; 207.33 ± 41.21  IL-8; 369.18 ± 111.34 | NS^*^  NS^†^ |

PEX; pseudoexfoliation syndrome, PEXG; pseudoexfoliation glaucoma, PSS; Posner-Schlossman syndrome, POAG; primary open angle glaucoma, NVG; neovascular glaucoma, FUS; Fuchs’ uveitis syndrome.

* Comparison of concentration of MCP-1 in aqueous humor of the diseased eye compared with controls.

† Comparison of concentration of IL-8 in aqueous humor of the diseased eye compared with controls.

n.s. = not statistically significant (*P* ≥ 0.05)

n.a = not accessible

**Table S2. Oligonucleotide primer sequence used in this study**

| Gene Name | Forward Primer | Reverse Primer | Product Size |
| --- | --- | --- | --- |
| *TGF-β1* | GAGCCTGAGGCCGACTACTA | GGGTTCAGGTACCGCTTCTC | 149bp |
| *TGF-β2* | GTCGCGCTCAGCCTGTCT | CCTCGATCCTCTTGCGCAT | 69bp |
| *MCP-1* | AGAATCACCAGCAGCAAGTGTCC | ATGGAATCCTGAACCCACTTCTGC | 107bp |
| *IL-8* | GACCACACTGCGCCAACAC | CTTCTCCACAACCCTCTGCAC | 106bp |
| *CCR2* | AGCAGGAAGATGAGGACAACAGCA | ACAATACAGCAAACTGGCGGATGC | 195bp |
| *CXCR1* | AGCCAGATCACCTTCCACACACAA | GCAAGGAGTTCTTGGCACGTCATT | 156bp |
| *α-SMA* | GACAATGGCTCTGGGCTCTGTAA | CTGTGCTTCGTCACCCACGTA | 146bp |
| *Fibronectin* | CTGGCCGAAAATACATTGTAA | CCACAGTCGGGTCAGGAG | 113bp |
| *FAK* | GTGCTCTTGGTTCAAGCTGGA | ACTTGAGTGAAGTCAGCAAGATGTG | 117bp |
| *PXN* | ACGTCTACAGCTTCCCCAACAA | AGCAGGCGGTCGAGTTCA | 99bp |
| *LAYN* | CACAGCCTGCCAGGACCTTTA | TGCACCGGTCATCATTCCA | 175bp |
| *FSP-1* | GCAACAGGGACAACGAGG | CTGGGCTGCTTATCTGGG | 105bp |
| HSV-1 DNA polymerase | CATCACCGACCCGGAGAGGGAC | GGGCCAGGCGCTTGTTGGTGTA | 91bp |
| CMV *UL26* | AACATCGCGTCGGTGATTTCTTGC | ACAGCTACTTTGAAGACGTGGAGC | 178bp |


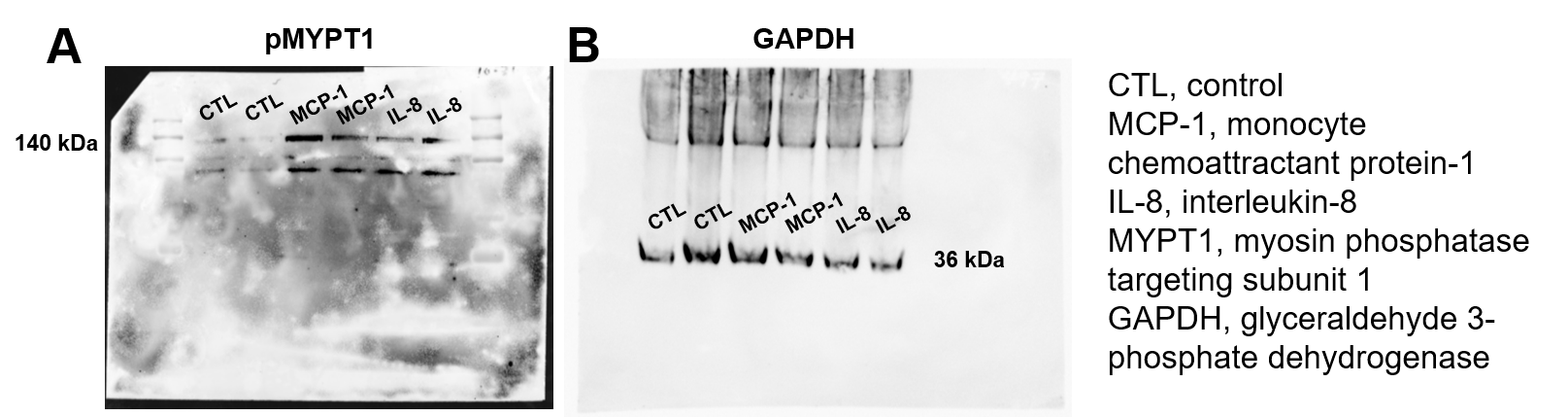


**Supplementary Figure S1. The full-length gels. The original gels reporting pMYPT1, and GAPDH expression in Fig 1B.**


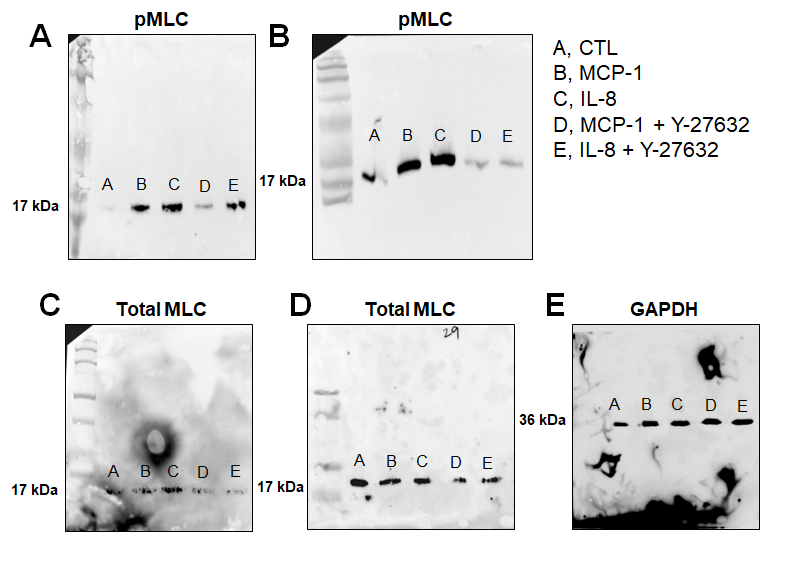


**Supplementary Figure S2. The full-length gels. The original gels reporting pMLC (myosin light chain kinase), total MLC and GAPDH expression in Fig 3A.**

**
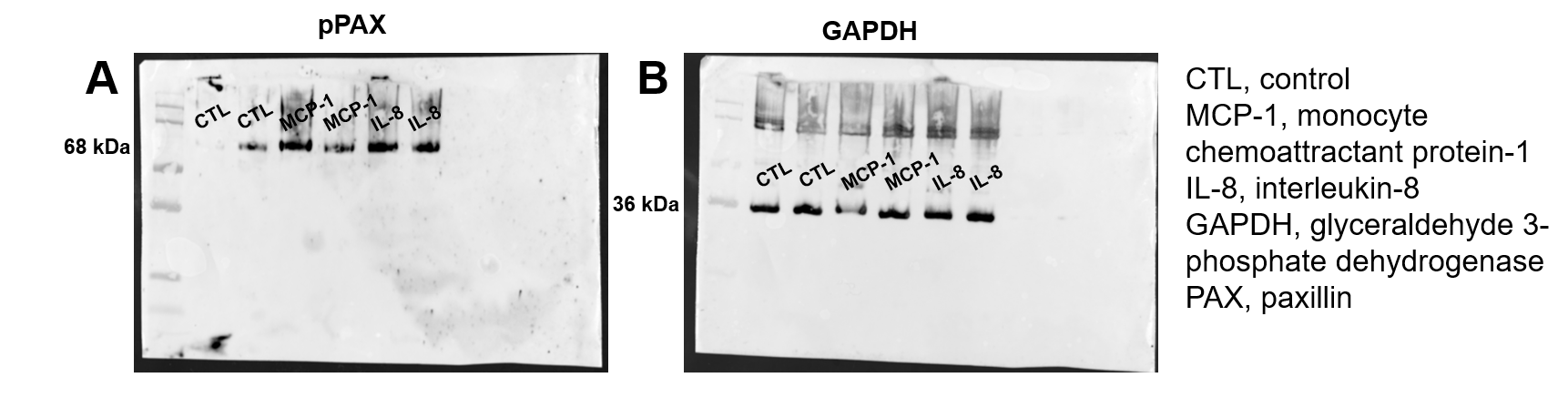
**

**Supplementary Figure S3. The full-length gels. The original gels reporting pPAX and GAPDH expression in Fig 4B.**

**
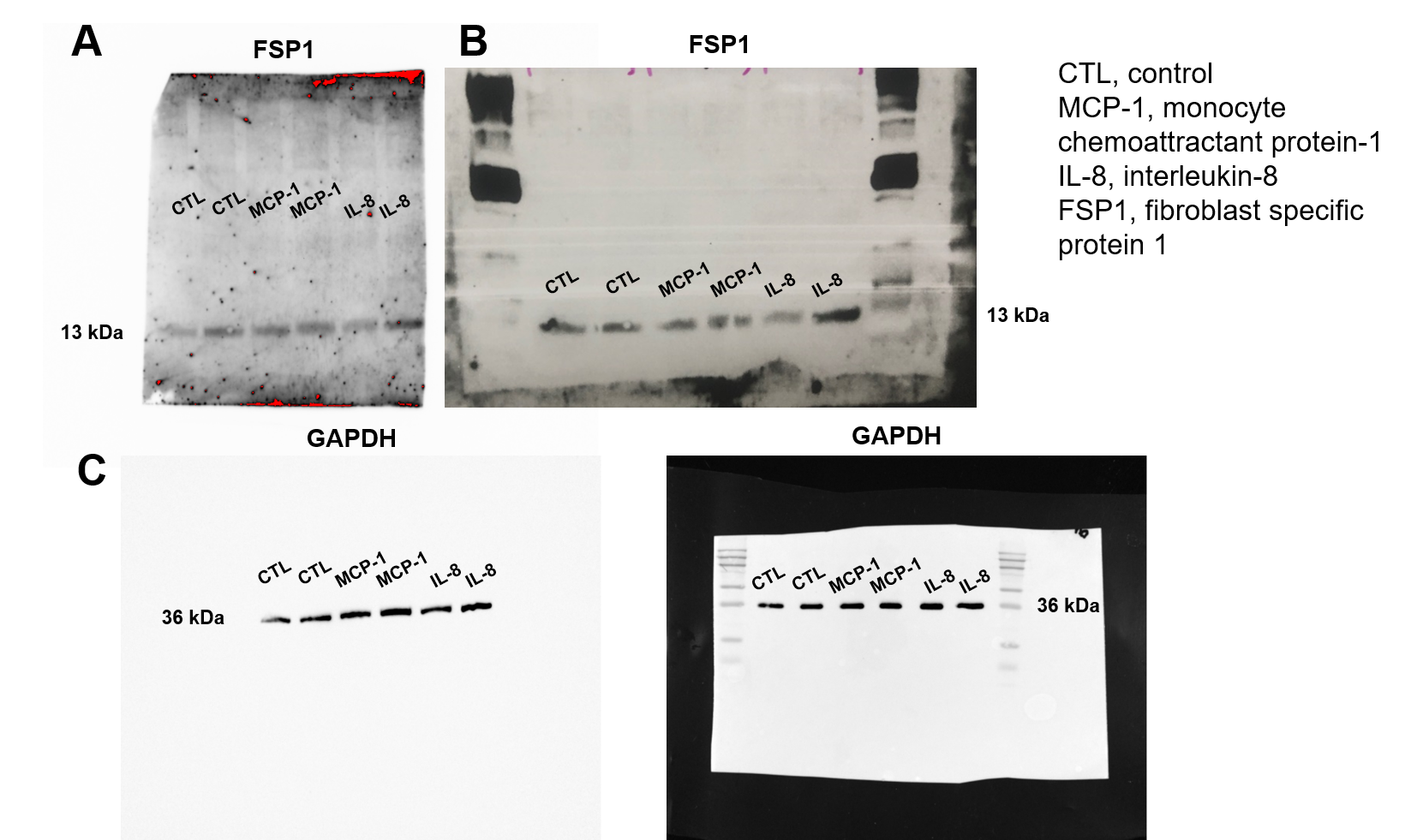
**

**Supplementary Figure S4. The full-length gels. The original gels showing FSP1 and GAPDH expression in Fig 5B.**


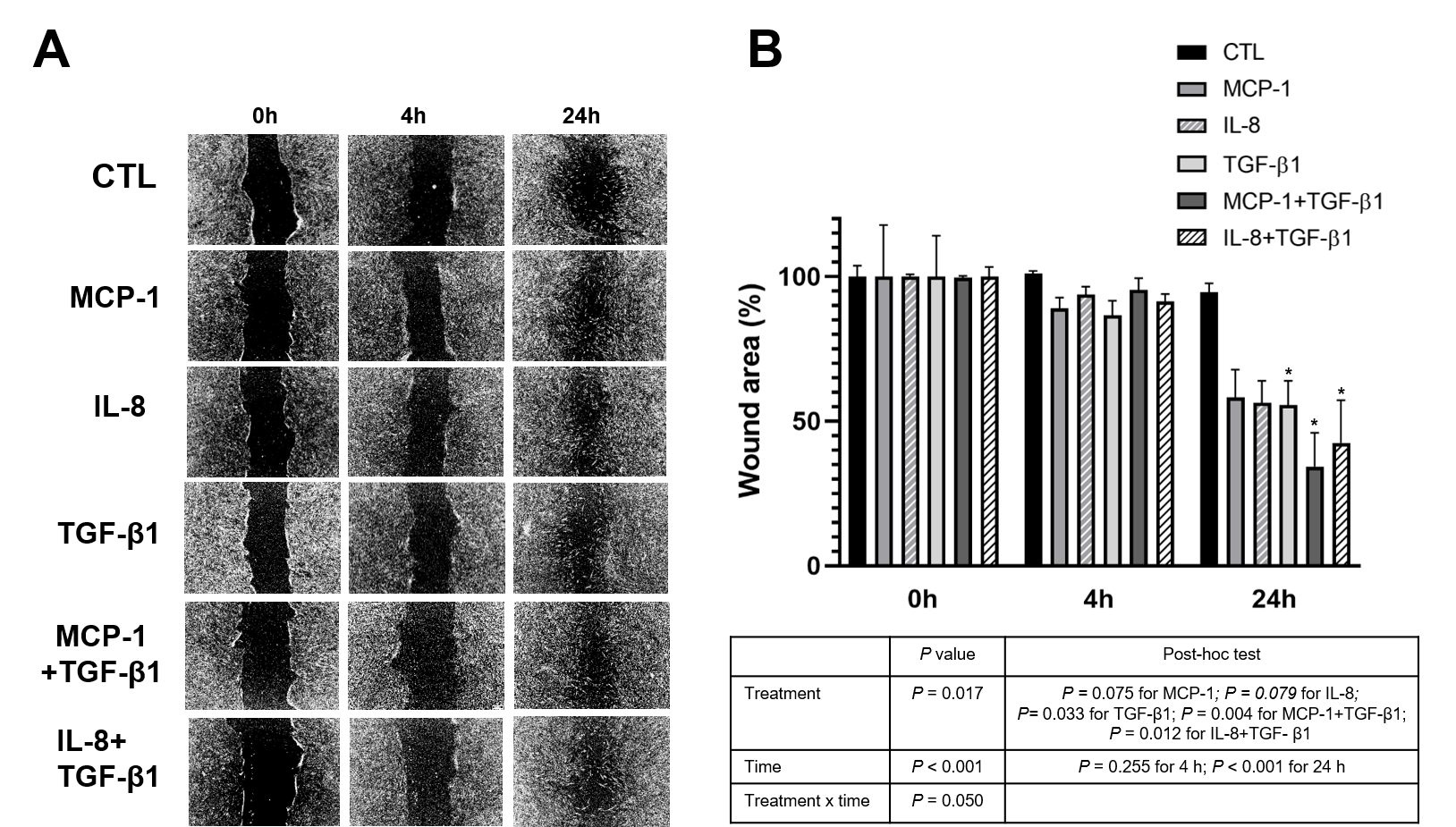


**Supplementary Figure S5. MCP-1 and IL-8 induces TM cell migration.** Sub-confluent monolayers of human primary TM cells obtained from ScienCell Research Labs were plated on 6 well plates. The monolayers were scratched with a pipette tip and incubated for 4h or 24 h with MCP-1 (100ng/ml) or IL-8 (100 ng/ml) in the presence or absence of TGF-β1 (15ng/ml). Serum-free medium was used as a negative control and TGF-β1 was used as a positive control. (A) Representative images demonstrating wound-induced TM cell migration at 4 h and 24 h. The scratch wound healing assay shows that MCP-1 and IL-8 significantly increased the migration of HTM cells, compared with to the control, which was further enhanced by the co-treatment with TGF-β1. (B) A bar chart representing the relative wound area per 20 x field. Cell migration was evaluated using Image J software. All results were based on three biological replicates. **P* ≤ 0.05 analyzed by two-way ANOVA with Dunnett’s multiple comparison.

**
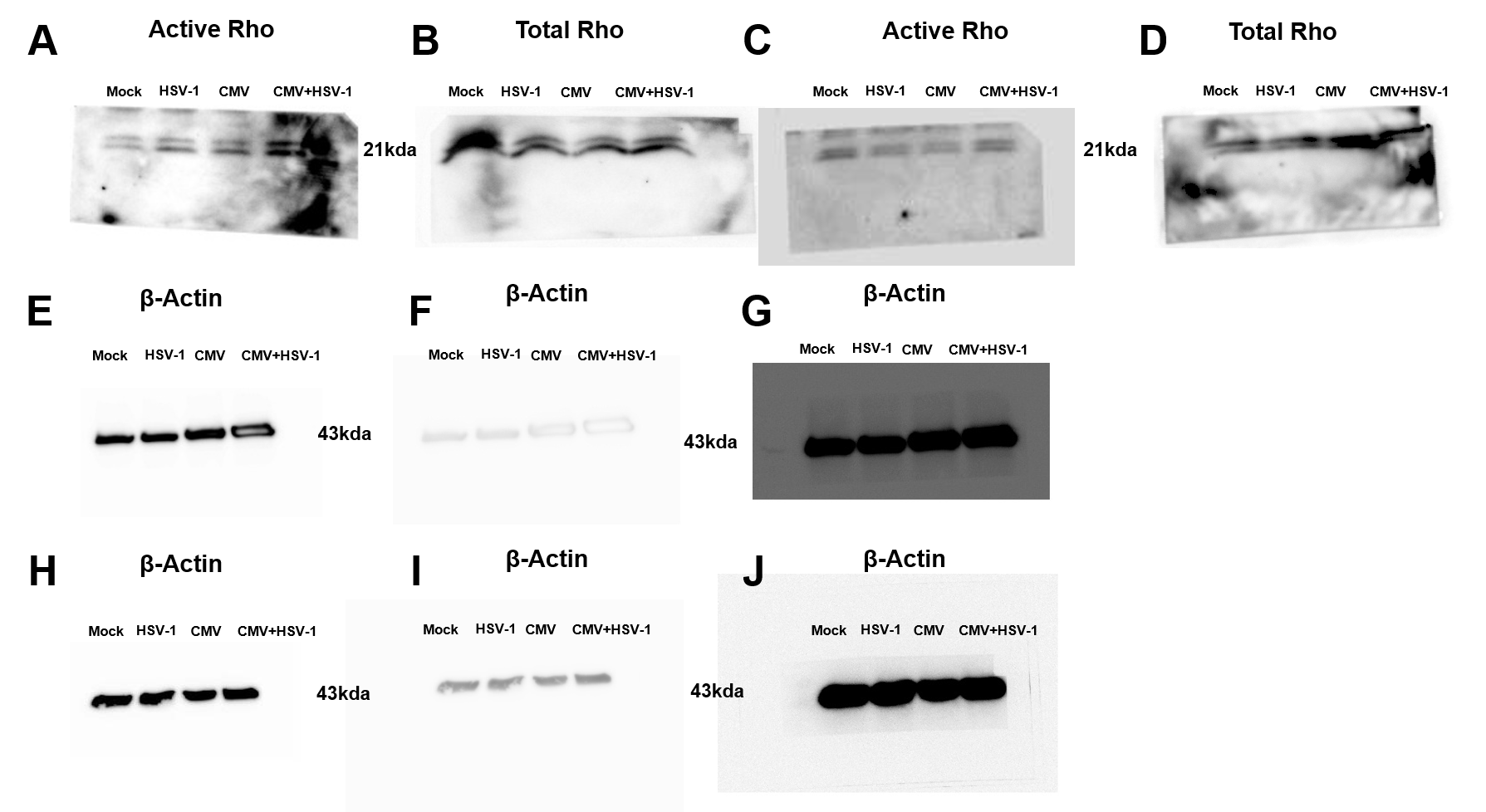
**

**Supplementary Figure S6. The full-length gels. The original gels showing active Rho (A,C), total Rho (B,D), and β-actin (E-J) expression in Fig 6K. Same blot with multiple exposures (E-G and H-J) is shown.**

**
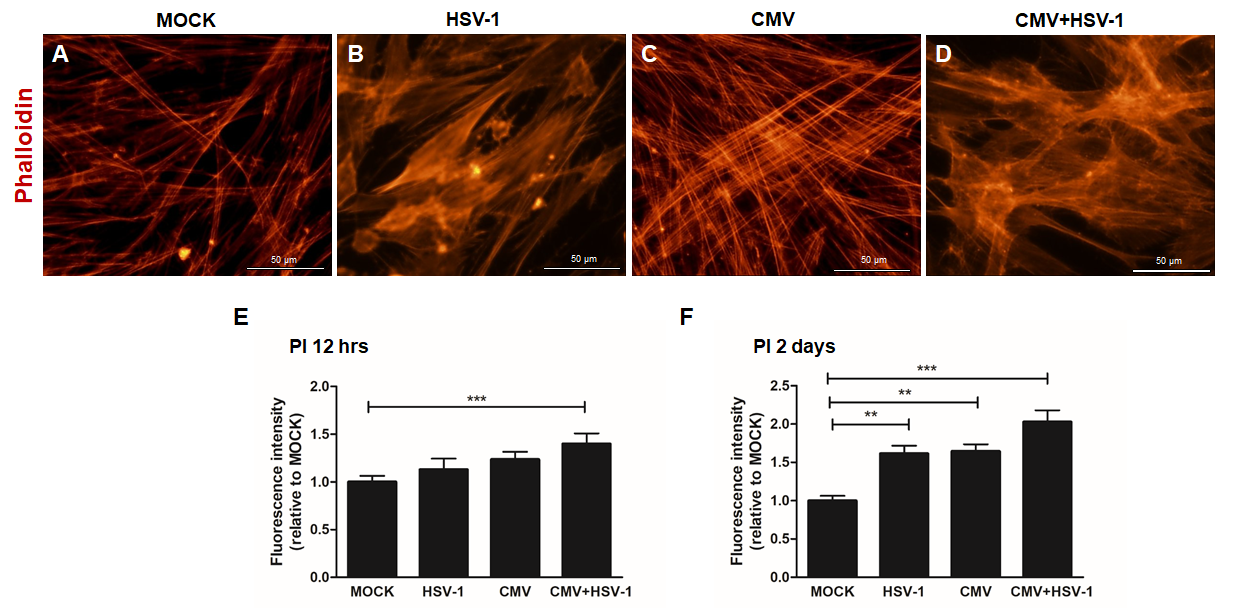
**

**Supplementary Figure S7. High magnification images of HSV-1 and CMV infection-induced changes in the human TM cell actin cytoskeletal organization.** Primary TM cells obtained from ScienCell Research Labs were infected with HSV-1 or CMV alone, or with HSV-1 and CMV at a multiplicity of infection of 1, and observed at 12 hours and 2 days post-infection (PI). High magnification images at 2 days PI (A,B,C,D) and the mean fluorescence intensity at 12 hours (E) and 2 days PI (F) are shown. The fluorescence intensity was measured as a relative arbitrary unit using ImageJ software under the same settings and conditions for each sample. Values are mean ± SEM, N = 3, ***P* ≤ 0.01; ****P* ≤ 0.001 using Dunnett's multiple comparison.


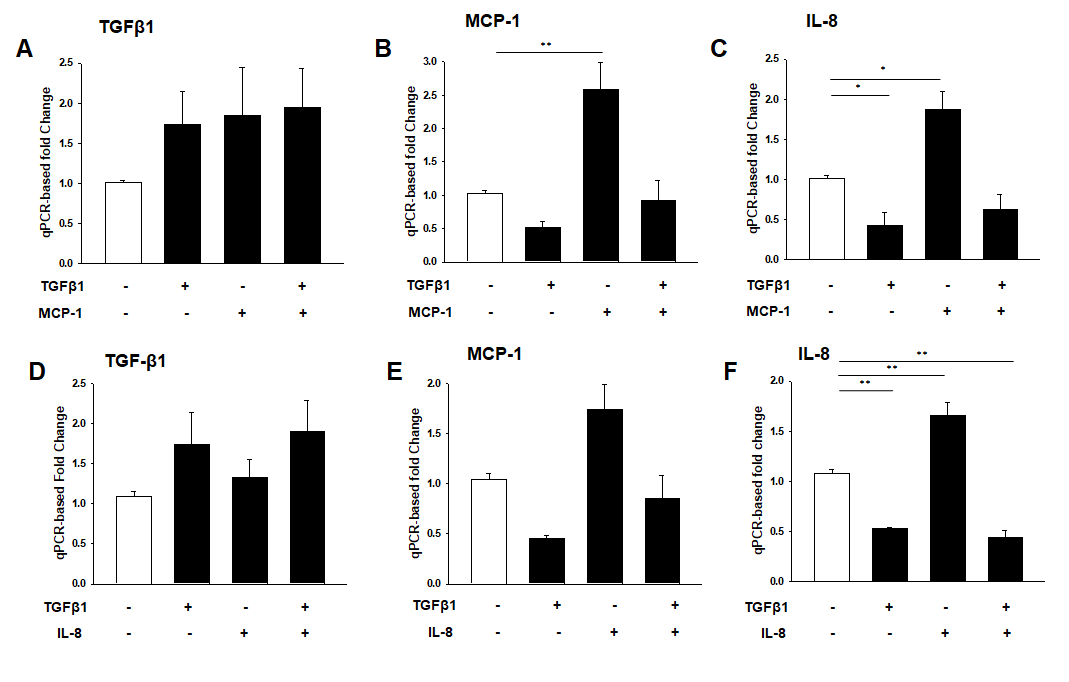


**Supplementary Figure S8. Quantification of interaction among MCP-1, IL-8 and TGF-β 1 in human TM cells using real-time qPCR analyses.** Serum-starved for 24 h were treated with MCP-1 (100 ng/ml) or IL-8 (100 ng/ml) for 2 h in the presence or absence of TGF-β1 (15 ng/ml) for 24 h. The expression of TGF-β 1 (A,D), MCP-1 (B,E) and IL-8 (C,F) upon treatment with MCP-1 (A-C) or IL-8 (D-F) in the presence or absence of TGF-β1. Data are shown as means ± SEM, N=3. **P < 0.05; **P < 0.01* calculated using one-way ANOVA with Dunnett’s multiple comparison.


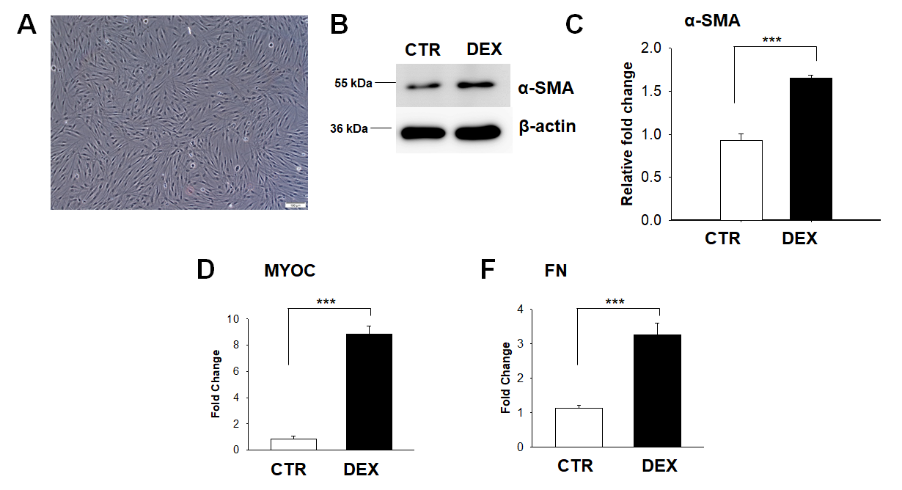


**Supplementary Figure S9. Characterization of primary TM cells obtained from ScienCell Research Labs.** The serum-starved TM cells subjected to the treatment with dexamethasone (DEX, 500 nM for 4 days). The confluent TM cells show a cobblestone-like pattern with some overlapping processes (A). Immunoblot analyses confirmed that the untreated control TM cells (CTR) exhibit the expression of α-smooth muscle actin (SMA), which is significantly increased upon the treatment with DEX (B). Histograms depict the fold change in the levels of α-SMA in the TM cells treated with DEX, based on densitometric analysis (C). The TM cells treated with DEX showed significantly increased expression of myocilin (MYOC) and fibronectin (FN) using real-time qPCR analyses (D,F). Values are mean ± SEM, N = 3, ****P* ≤ 0.001 (Student’s *t*-test).


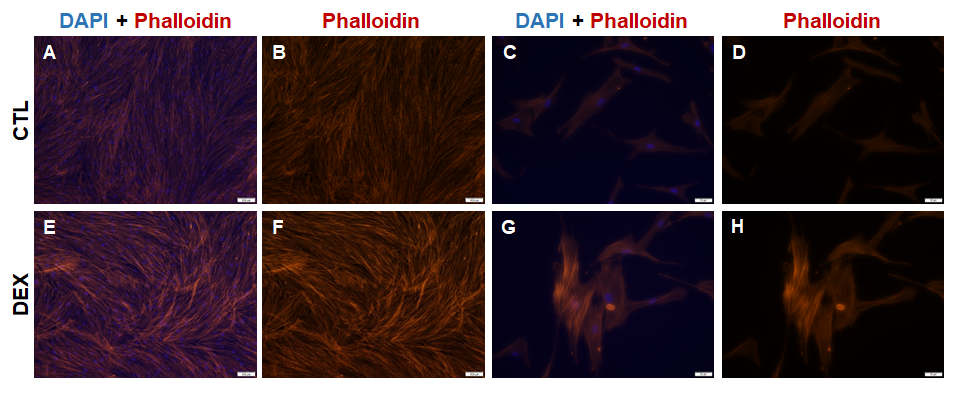


**Supplementary Figure S10. Changes in the human TM cell actin cytoskeletal organization with the dexamethasone (DEX) treatment.** The serum-starved primary TM cells obtained from ScienCell Research Labs were subjected to the treatment with DEX (500 nM for 4 days). Nuclei were stained with DAPI (blue signals) and stress fibers were stained with a Rhodamine Phalloidin (red signals) and observed in low (A,B,E,F) and high magnification (C,D,G,H). Significantly increased contraction of F-actin was observed with the DEX treatment in low (E,F) and high magnification (G,H), compared with controls (CTR) (A,B,C,D).
